# Supplementary material for: A protein-specific priority code in presequences determines the efficiency of mitochondrial protein import
Source: PLoS Biol. 2025 Jul 21;23(7):e3003298. doi: 10.1371/journal.pbio.3003298 (PMC12306757; doi:10.1371/journal.pbio.3003298)
Supplement: S1 Fig — (A) Schematic visualization of a multi-step workflow for LLM-based physiochemical embedding, created to compare different MTS in silico. The workflow begins by replacing the N-terminal methionine of the cytosolic protein dihydrofolate reductase (DHFR) with the targeting sequences. The engineered proteins are then processed through a protein language model to embed the sequences in high-dimensional space. Subsequently, the DHFR sequence is cleaved off, leaving only the embedded MTS sequence. To mitigate any bias related to sequence length, a mean-pooling layer is applied. Finally, Uniform Manifold Approximation and Projection (UMAP) is used to project the embedded sequences into two-dimensional space for interpretation. This workflow is repeated for each MTS, with UMAP applied to all embedded sequences simultaneously to facilitate grouping of similar vectors. (B) Evolutionary Comparison of MTS Across Species. Comparison of clusters identified in the S. cerevisiae datasets (220 MTS—colored) with an expanded dataset containing 1,450 MTS from proteins of a comprehensive set of organisms (S1 Table) reveals the evolutionary robustness of the different groups found in yeast. (C) Shown are the consistencies of changes upon different perturbations for representatives of the different groups of presequences. The absolute mean values of the changes within a group were calculated. The significance indicates how likely it is to obtain the respective mean value by chance, considering the cluster size, the respective condition, and the respective cluster. Thus, high values mean that proteins with specific presequence types are more consistently affected than it would be expected if the distribution was random. The data underlying the graphs shown in the figure can be found in S1 Data. (PDF) [file pbio.3003298.s001.pdf]

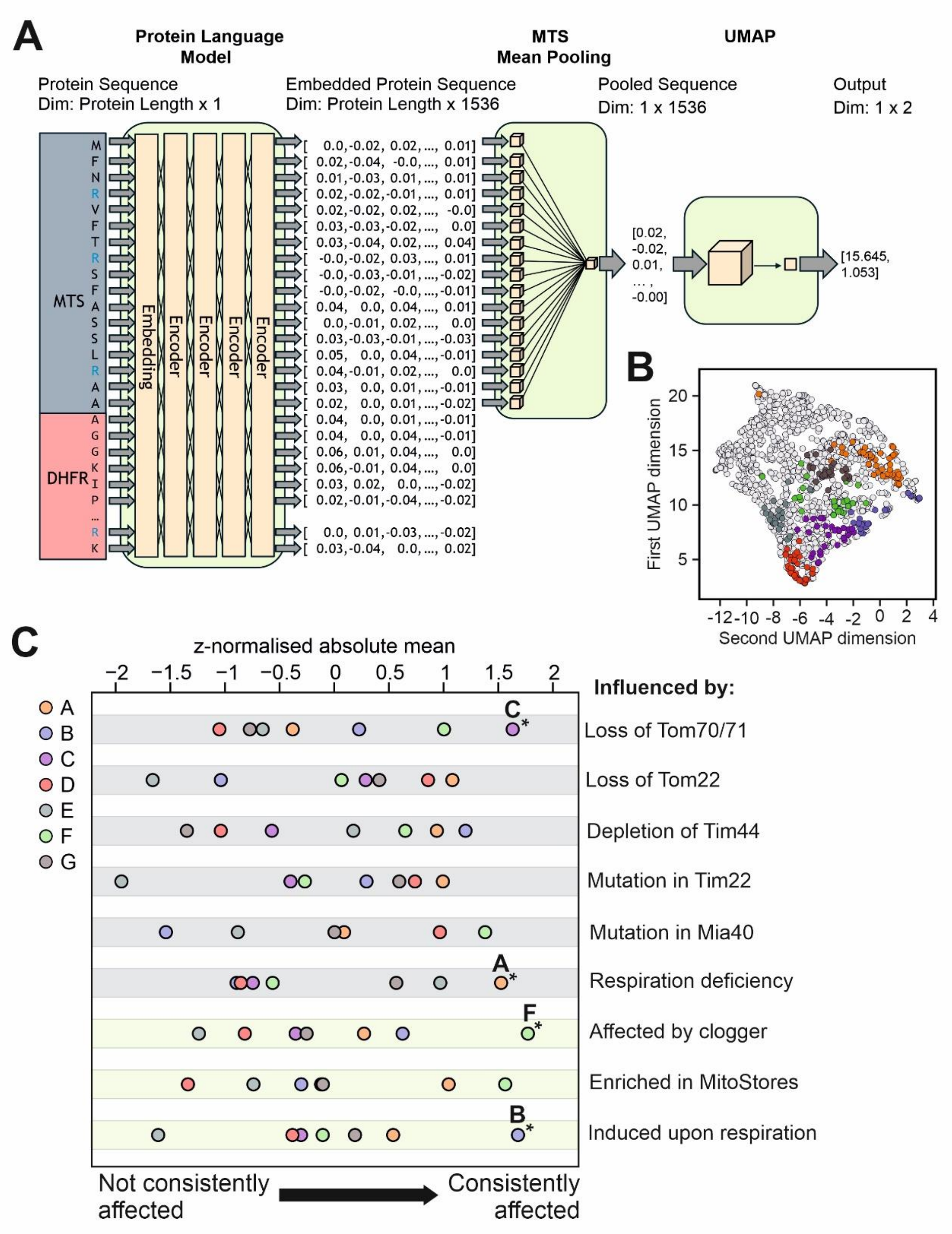

Fig S1: Classification of presequences

(A) Schematic visualization of a multi-step workflow for LLM-based physiochemical embedding, created to compare different MTS in silico. The workflow begins by replacing the N-terminal methionine of the cytosolic protein dihydrofolate reductase (DHFR) with the targeting sequences. The engineered proteins are then processed through a protein language model to embed the sequences in high-dimensional space. Subsequently, the DHFR sequence is cleaved off, leaving only the embedded MTS sequence. To mitigate any bias related to sequence length, a mean-pooling layer is applied. Finally, Uniform Manifold Approximation and Projection (UMAP) is used to project the embedded sequences into two-dimensional space for interpretation. This workflow is repeated for each MTS, with UMAP applied to all

embedded sequences simultaneously to facilitate grouping of similar vectors. (B) Evolutionary Comparison of MTS Across Species. Comparison of clusters identified in the *S. cerevisiae datasets* (220 MTS - colored) with an expanded dataset containing 1450 MTS from proteins of a comprehensive set of organisms (S1_Table) reveals the evolutionary robustness of the different groups found in yeast. (C) Shown are the consistencies of changes upon different perturbations for representatives of the different groups of presequences. The absolute mean values of the changes within a group were calculated. The significance indicates how likely it is to obtain the respective mean value by chance, considering the cluster size, the respective condition, and the respective cluster. Thus, high values mean that proteins with specific presequence types are more consistently affected than it would be expected if the distribution was random. The data underlying the graphs shown in the figure can be found in S1_Data.
