# Supplementary material for: A protein-specific priority code in presequences determines the efficiency of mitochondrial protein import
Source: PLoS Biol. 2025 Jul 21;23(7):e3003298. doi: 10.1371/journal.pbio.3003298 (PMC12306757; doi:10.1371/journal.pbio.3003298)
Supplement: S2 Fig — List of the gene names that correspond to the embedded MTS sorted according to the identified cluster. (PDF) [file pbio.3003298.s002.pdf]

	A	B	C	D	E	F	G
Enzymes	Acp1<br>Ald4<br>Atp1<br>Atp14<br>Atp16<br>Coq2<br>Cox6<br>Cox11<br>Cox15<br>Edh3<br>Gcv2<br>Hem1<br>Ilv2<br>Ilv5<br>Lip2<br>Mae1<br>Ndi1<br>Nfs1<br>Pdb1<br>Pdx1<br>Psd1<br>Sco2<br>Sdh1<br>Sdh3<br>Sdh4<br>Ysa1	Atp5<br>Cit3<br>Cor1<br>Cox4<br>Cpr3<br>Ecm31<br>Icp55<br>Idp1<br>Inh1<br>Lpd1<br>Lsc1<br>Lys4<br>Mdh1<br>Stf1	Aco1<br>Ald5<br>Atp17<br>Cox5a<br>Idh2<br>Lat1<br>Lys12<br>Mcr1<br>Qcr2<br>Rip1<br>Shm1	Aat1<br>Acn9<br>Arh1<br>Atp6<br>Bat1<br>Coq5<br>Cyc2<br>Etr1<br>Glo4<br>Icl2<br>Idh1<br>Leu9<br>Lsp1<br>Nfu1<br>Put2<br>Rnr3<br>Sdh2<br>Ymr31	Atp11<br>Atp12<br>Atp25<br>Coq10<br>Emi5<br>Yor356w	Abc1<br>Atp3<br>Cit1<br>Cox8<br>Dld2<br>Fum1<br>Grx5<br>Ilv6<br>Isa2<br>Kgd1<br>Lip5<br>Lsc2<br>Mis1<br>Nde1	Alt1<br>Arg5,6<br>Atp15<br>Bna3<br>Cbp4<br>Ccp1<br>Cyt1<br>Gut2<br>Isu1<br>Kgd2<br>Nem1<br>Ppa2<br>Tdh3
Biogenesis	Mam33<br>Mba1<br>Mge1<br>Mia40<br>Oxa1<br>Pim1<br>Tim44	Hsp60	Afg1<br>Mas1<br>Tcm62	Cym1<br>Mas2<br>Mcx1<br>Phb2<br>Yme1	Oct1<br>Pam16	Afg3<br>Tim21	
MRPs	Mrpl22<br>Mrps28	Mrp21<br>Mrpl6<br>Mrps5<br>Mrps9<br>Rsm22	Img1<br>Mrpl1<br>Mrpl15<br>Rsm9<br>Rsm28	Mrp1<br>Mrp2<br>Mrpl36<br>Rsm26<br>Yml6	Mrp20<br>Mrp7<br>Mrpl11<br>Mrpl16<br>Mrpl27<br>Mrpl50<br>Mrps17<br>Nam9<br>Rsm24	Mrpl24	Img2<br>Mrp13<br>Mrpl10<br>Mrpl28
others	Fmp16<br>Fmp29<br>Mdl2<br>Mef1<br>Mmt2<br>Msc6<br>Mss116<br>Prx1<br>Tuf1<br>Ygr031w<br>Ynr036c	Fmp23<br>Fmp26<br>Rim1<br>Yir100c	Abf2<br>Fmp12<br>Fmp31<br>Gcv3<br>Gif1<br>Mgm101<br>Yir003c<br>Yme2<br>Yor215c	Fmp10<br>Msf1<br>Nif3<br>Por2<br>Utp10<br>Yjl133c-a<br>Ypr004c	Dis3<br>Fmp34<br>Msk1<br>Mss51<br>Rex2	Cat2<br>Fmp24<br>Fmp32<br>Fmp39<br>Mrs2<br>Msy1<br>Mtf2<br>Sco1	Aep2<br>Dss1<br>Fmp30<br>Fun14<br>Gnp1<br>Iml2<br>Ism1<br>Kip2<br>Mmt1<br>Pkp1<br>Pma1<br>Pom152<br>Rmd9<br>Rpm2<br>Trx3<br>Yel067c<br>Yer087w<br>Yer140w<br>Ygl059w<br>Ygr150c<br>Ynl213c<br>Yor022c

Fig S2: Presequences can be sorted into seven distinct groups on basis of their sequence

List of the gene names that correspond to the embedded MTS sorted according to the identified cluster.
