# Supplementary material for: A protein-specific priority code in presequences determines the efficiency of mitochondrial protein import
Source: PLoS Biol. 2025 Jul 21;23(7):e3003298. doi: 10.1371/journal.pbio.3003298 (PMC12306757; doi:10.1371/journal.pbio.3003298)
Supplement: S3 Fig — (A–C) Mitochondria were isolated from wild type, ∆cox18, and ∆atp6 mutants. The ∆atp6 strain was grown in the absence of arginine to ensure the presence of the mitochondrial genome. This mutant contained the ARG8 gene at the position of the mitochondrially encoded ATP6 locus [76]. For panel C, the strain MR6 was used as a corresponding wild type of the ∆atp6 mutant. The indicated proteins were radiolabeled and imported as described for Fig 2A. Import efficiencies were quantified and are shown in relation to the import efficiency into wild-type mitochondria. The data underlying the graphs shown in the figure can be found in S1 Data. (PDF) [file pbio.3003298.s003.pdf]

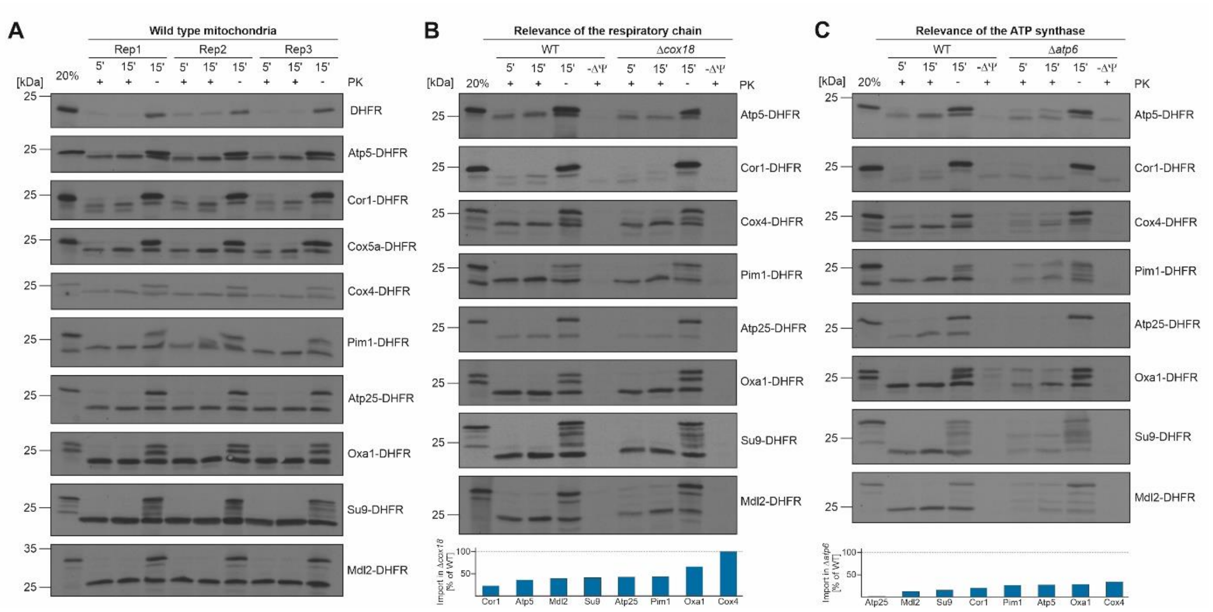

Fig S3: All tested presequences depend on the levels of the membrane potential and ATP

(A-C) Mitochondria were isolated from wild type, Δ*cox18* and Δ*atp6* mutants. The Δ*atp6* strain was grown in the absence of arginine to ensure the presence of the mitochondrial genome. This mutant contained the *ARG8* gene at the position of the mitochondrially encoded *ATP6* locus [73]. For panel C, the strain MR6 was used as a corresponding wild type of the Δ*atp6* mutant. The indicated proteins were radiolabeled and imported as described for Fig 2A. Import efficiencies were quantified and are shown in relation to the import efficiency into wild type mitochondria. The data underlying the graphs shown in the figure can be found in S1_Data.
