# Supplementary material for: A protein-specific priority code in presequences determines the efficiency of mitochondrial protein import
Source: PLoS Biol. 2025 Jul 21;23(7):e3003298. doi: 10.1371/journal.pbio.3003298 (PMC12306757; doi:10.1371/journal.pbio.3003298)
Supplement: S4 Fig — (A) Scheme of the in vivo import assay IQ-Compete. (B, F) Microscopy images of the respective strains. See legend to Fig 3D for details. (C, D) Fluorescence intensities of the depicted strains were measured using a multiplate fluorescence spectrometer (Clariostar, BMG Labtech). Shown are mean values and standard deviations of three biological replicates. (E) The western blot of the samples shown in Fig 4B was probed with antibodies against DHFR for detection of the uTEV-DHFR fusion proteins. The data underlying the graphs shown in the figure can be found in S1 Data. (PDF) [file pbio.3003298.s004.pdf]

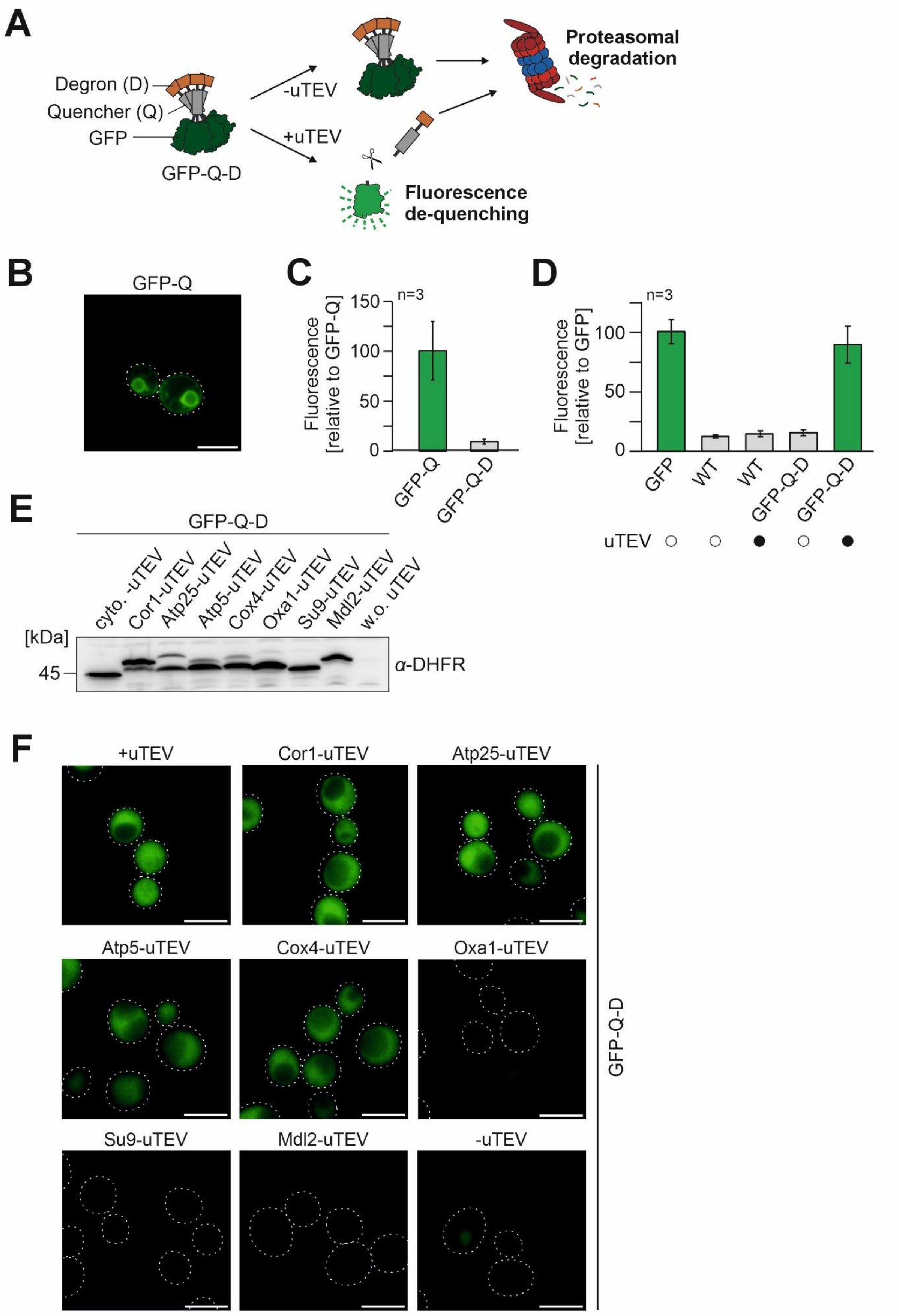
A
Degron (D)
Quencher (Q)
GFP
GFP-Q-D
-uTEV
+uTEV
Proteasomal degradation
Fluorescence de-quenching
B
GFP-Q
C
n=3
Fluorescence [relative to GFP-Q]
150
100
50
0
GFP-Q
GFP-Q-D
D
n=3
Fluorescence [relative to GFP]
100
50
0
GFP
WT
WT
GFP-Q-D
GFP-Q-D
uTEV
E
GFP-Q-D
cyto. -uTEV
Cor1-uTEV
Atp25-uTEV
Atp5-uTEV
Cox4-uTEV
Oxa1-uTEV
Su9-uTEV
Mdl2-uTEV
w.o. uTEV
[kDa]
45
α-DHFR
F
+uTEV
Cor1-uTEV
Atp25-uTEV
Atp5-uTEV
Cox4-uTEV
Oxa1-uTEV
Su9-uTEV
Mdl2-uTEV
-uTEV
GFP-Q-D

**Fig S4: Fluorescence intensities of the IQ-Comp assay can be measured by fluorescence spectroscopy and microscopy**

(A) Scheme of the *in vivo* import assay IQ-Compete. (B, F) Microscopy images of the respective strains. See legend to Fig 3D for details. (C, D) Fluorescence intensities of the depicted strains were measured using a multiplate fluorescence spectrometer (Clariostar, BMG Labtech). Shown are mean values and standard deviations of three biological replicates. (E) The Western blot of the samples shown in Fig 4B was probed with antibodies against DHFR for detection of the uTEV-DHFR fusion proteins. The data underlying the graphs shown in the figure can be found in S1_Data.
