# Supplementary material for: A protein-specific priority code in presequences determines the efficiency of mitochondrial protein import
Source: PLoS Biol. 2025 Jul 21;23(7):e3003298. doi: 10.1371/journal.pbio.3003298 (PMC12306757; doi:10.1371/journal.pbio.3003298)
Supplement: S5 Fig — (A, B) Su9-DHFR was imported into isolated mitochondria with or without urea-denaturation as described for Fig 5A and 5B. (C) Protein enrichment in Su9-DHFR samples relative to the DHFR control. See Fig 6B for details. (D) Specific enrichment (log2-fold change) of specific chaperones with Atp5-DHFR and Oxa1-DHFR relative to the DHFR control. (E) Coomassie-stained gels showing N-GST-TOMM34 expression in Escherichia coli under uninduced (−IPTG) and induced (+IPTG) conditions. Protein solubility was assessed by separating soluble (S) and insoluble (P) fractions. Recombinant GST-TOMM34 was purified using GSTrap chromatography, with flow-through (FT) and elution fractions shown. (F) Peptides of 20 residues representing the presequences of the indicated proteins were spotted onto a cellulose membrane. The individual peptide sequences were moved by a three-residue window (1-20, 4-23, 7-26, etc.). The membrane was incubated with purified recombinant GST-TOMM34. The membrane was washed, blocked with milk powder, and probed with an anti-GST antibody. (G) Model depicting the position of specific TOMM34 binding sites in presequences. The data underlying the graphs shown in the figure can be found in S1 Data. (PDF) [file pbio.3003298.s005.pdf]

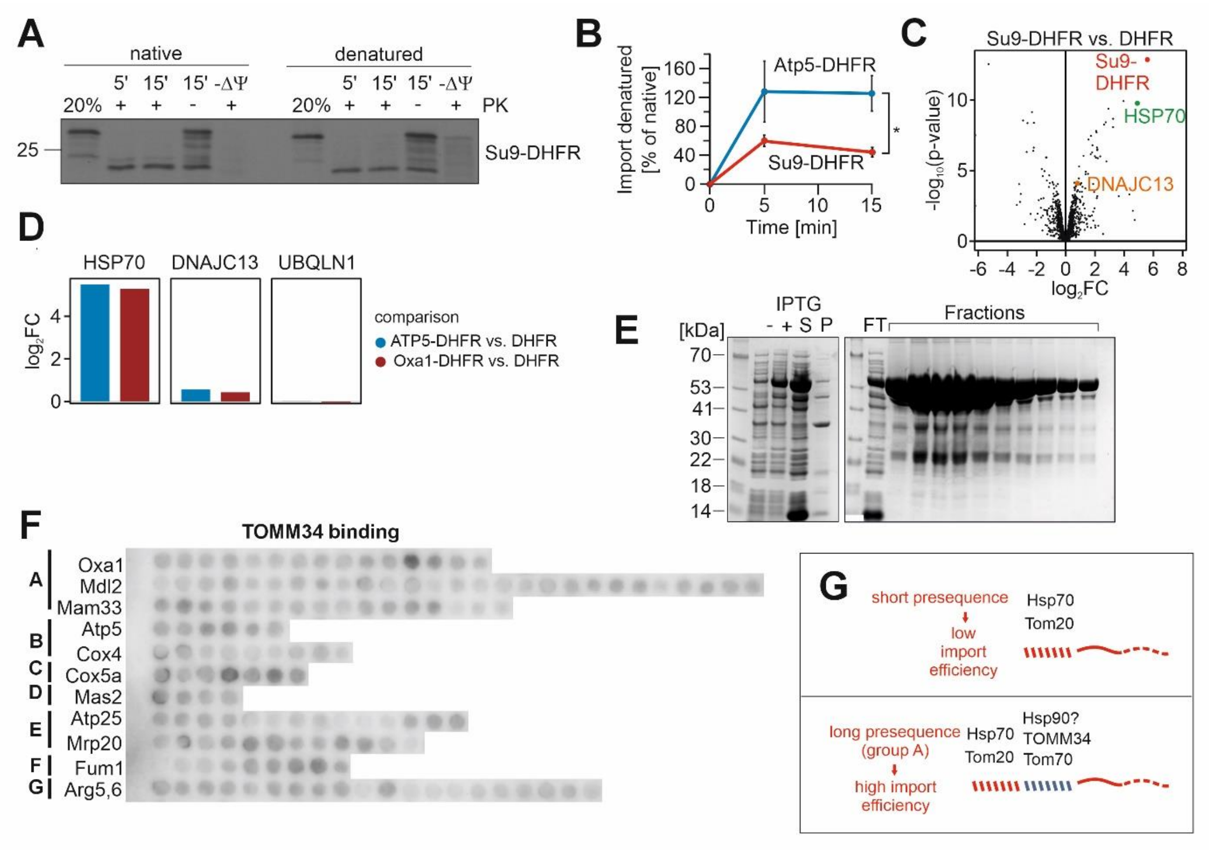

Fig S5: Presequences mediate the association with the cytosolic chaperone TOMM34

(A, B) Su9-DHFR was imported into isolated mitochondria with or without urea-denaturation as described for Fig. 5A and B. (C) Protein enrichment in Su9-DHFR samples relative to the DHFR control. See Fig. 6B for details. (D) Specific enrichment (log_2-fold change) of specific chaperones with Atp5-DHFR and Oxa1-DHFR relative to the DHFR control. (E) Coomassie-stained gels showing N-GST-TOMM34 expression in *E. coli* under uninduced (-IPTG) and induced (+IPTG) conditions. Protein solubility was assessed by separating soluble (S) and insoluble (P) fractions. Recombinant GST-TOMM34 was purified using GSTrap chromatography, with flow-through (FT) and elution fractions shown. (F) Peptides of 20 residues representing the presequences of the indicated proteins were spotted onto a cellulose membrane. The individual peptide sequences were moved by a three-residue window (1-20, 4-23, 7-26 etc.). The membrane was incubated with purified recombinant GST-TOMM34. The membrane was washed, blocked with milk powder and probed with an anti-GST antibody. (G) Model depicting the position of specific TOMM34 binding sites in presequences. The data underlying the graphs shown in the figure can be found in S1_Data.
