# Supplementary material for: A protein-specific priority code in presequences determines the efficiency of mitochondrial protein import
Source: PLoS Biol. 2025 Jul 21;23(7):e3003298. doi: 10.1371/journal.pbio.3003298 (PMC12306757; doi:10.1371/journal.pbio.3003298)
Supplement: S6 Fig — (A) Principal component analysis of the proteome data of the indicated strains. PC1 impressively shows the strong effect of Cns1 depletion which is not compensated by TOMM34 expression (PC2). (B) List of most severely depleted mitochondrial proteins in Cns1-depleted cells. (C, D) The expression of TOMM34 in the Cns1-depleted cells has only a very minor effect on mitochondrial proteins. The data underlying the graphs shown in panes A, C, and D can be found in S4 Table. (E) The expression of TOMM34 does not complement the growth defect of Cns1-depleted cells, whereas the expression of Cns1 from a plasmid complements the mutant. (PDF) [file pbio.3003298.s006.pdf]

<u>Raw Data</u>

Figure 2

Figure 2A

DHFR

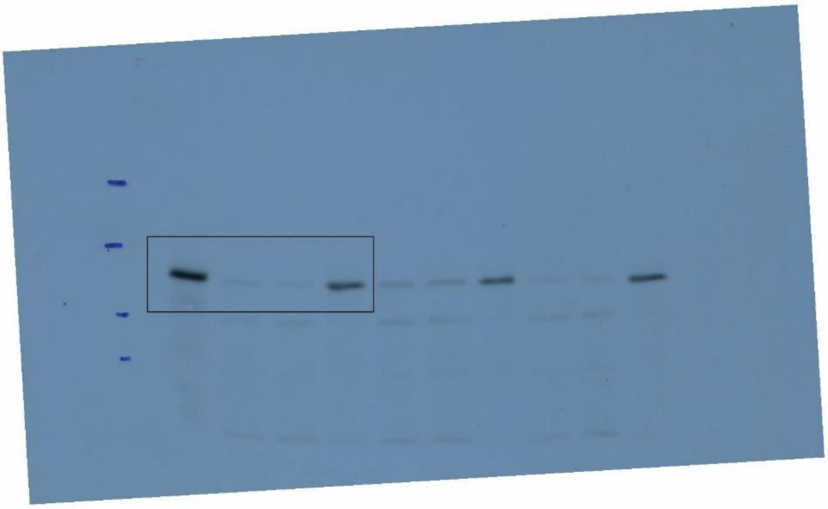

Cor1-DHFR

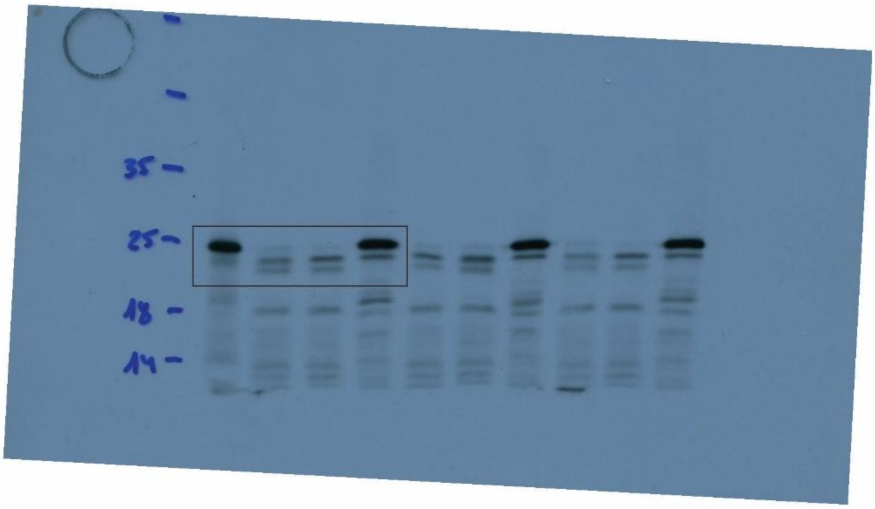

Cox5a-DHFR

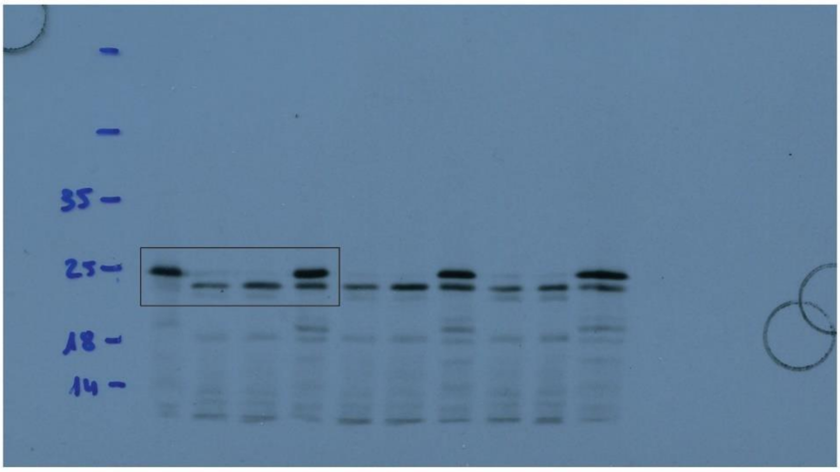

Cox4-DHFR

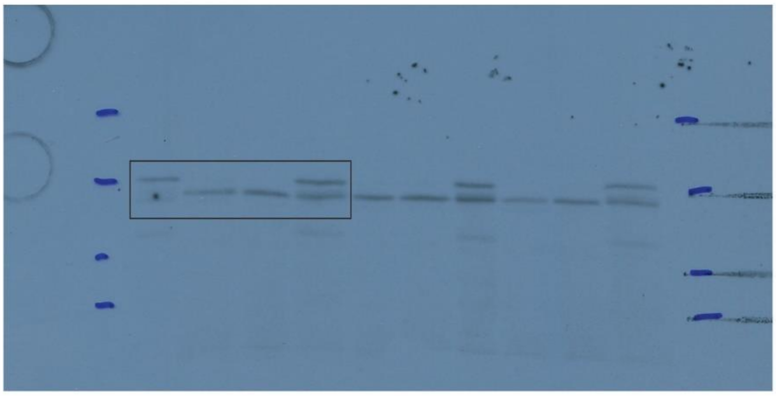

Atp5-DHFR

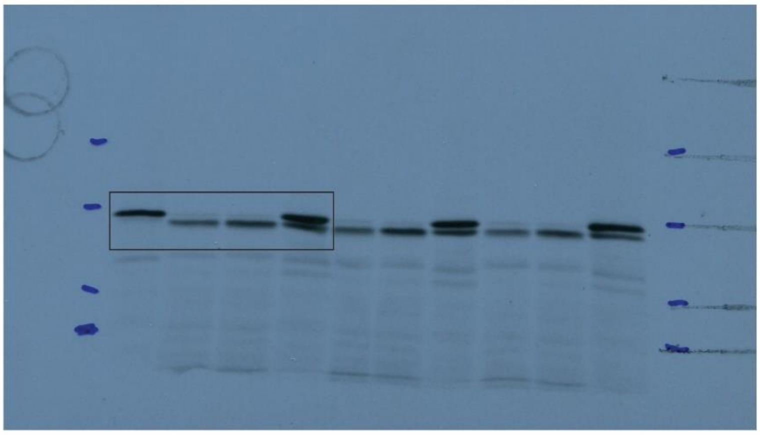

Atp25-DHFR

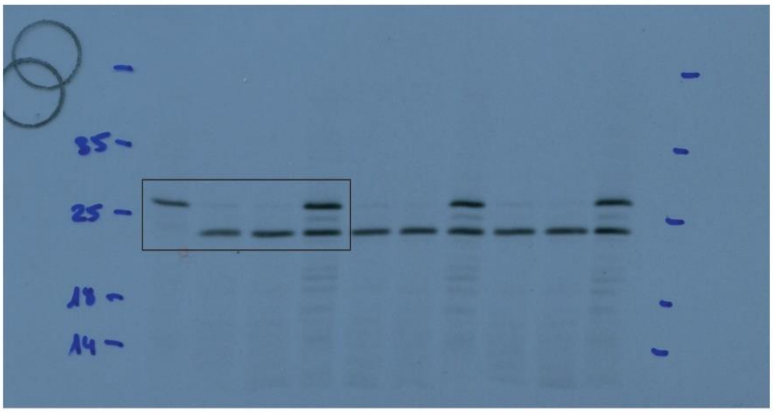

Pim1-DHFR

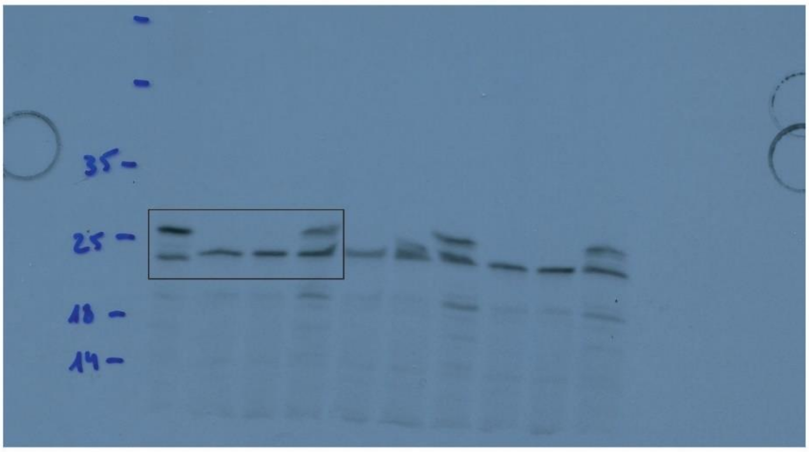

Su9-DHFR

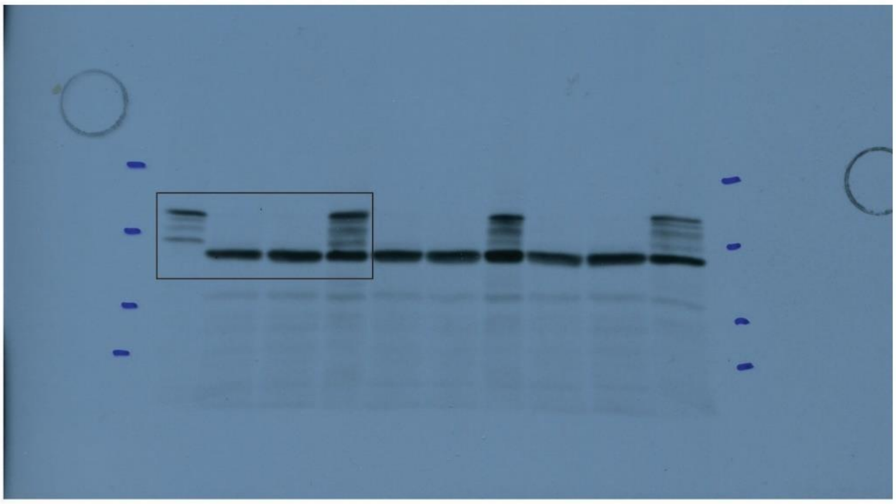

Mdl2-DHFR

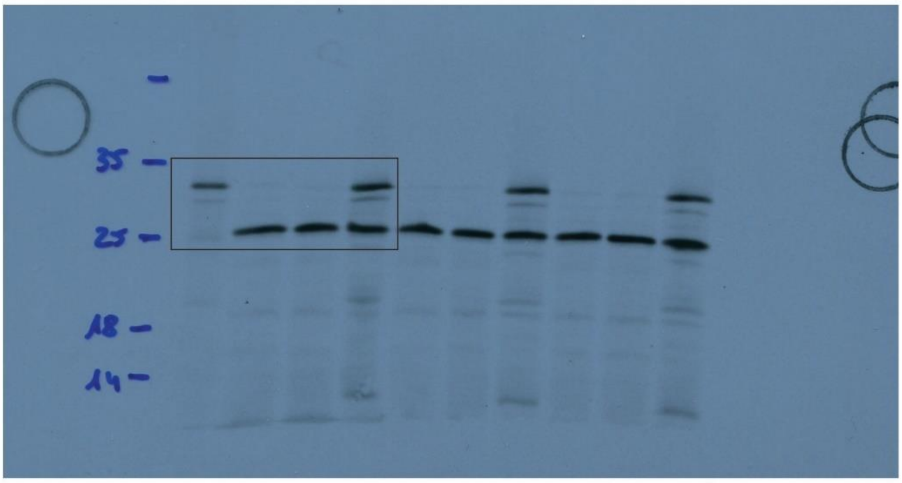

Oxa1-DHFR

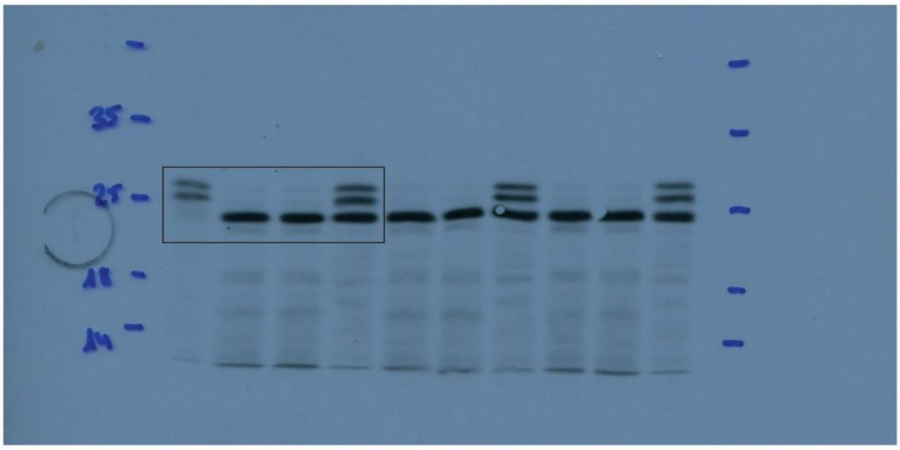

Figure 2B and S3A

Cox5a-DHFR

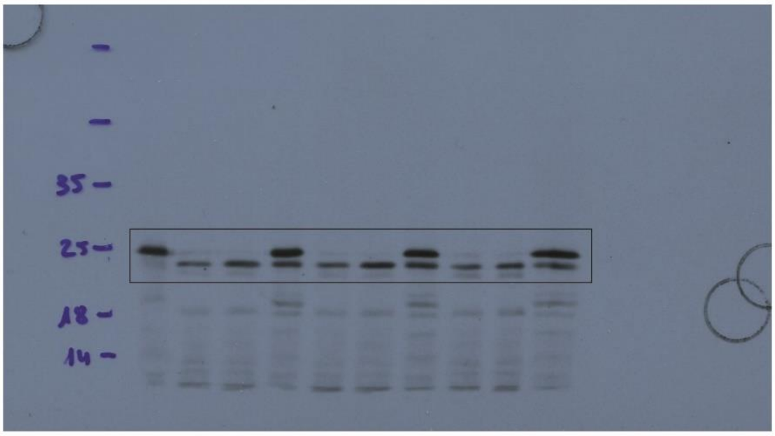

Cox4-DHFR

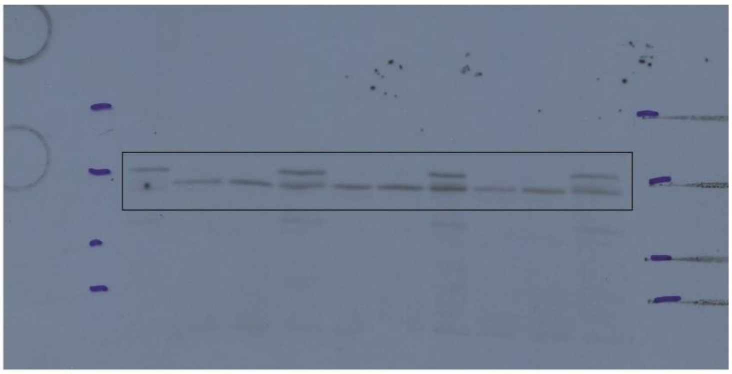

Pim1-DHFR

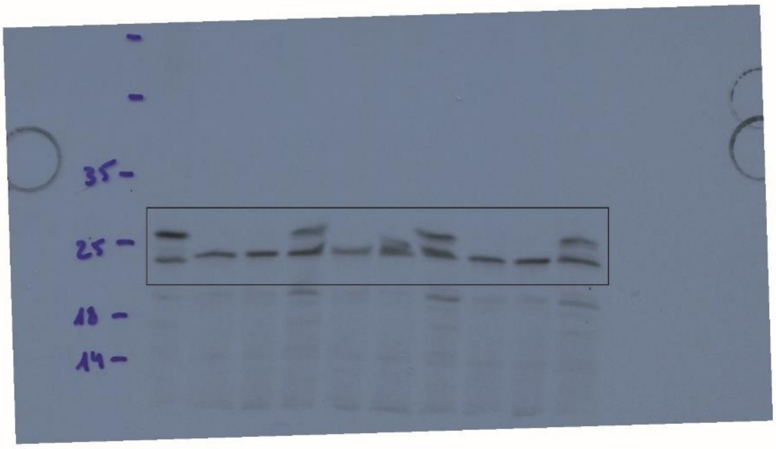

Atp25-DHFR

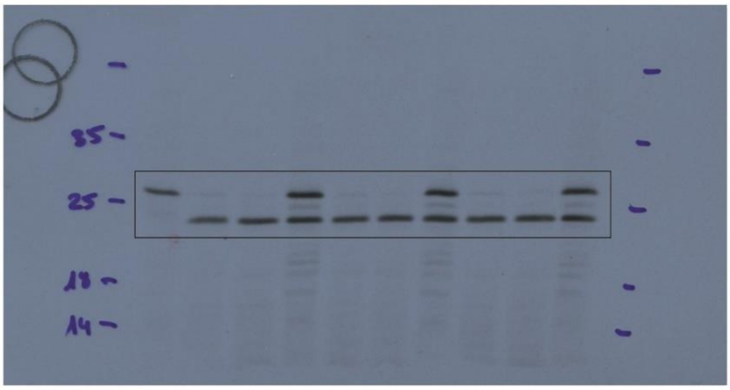

DHFR

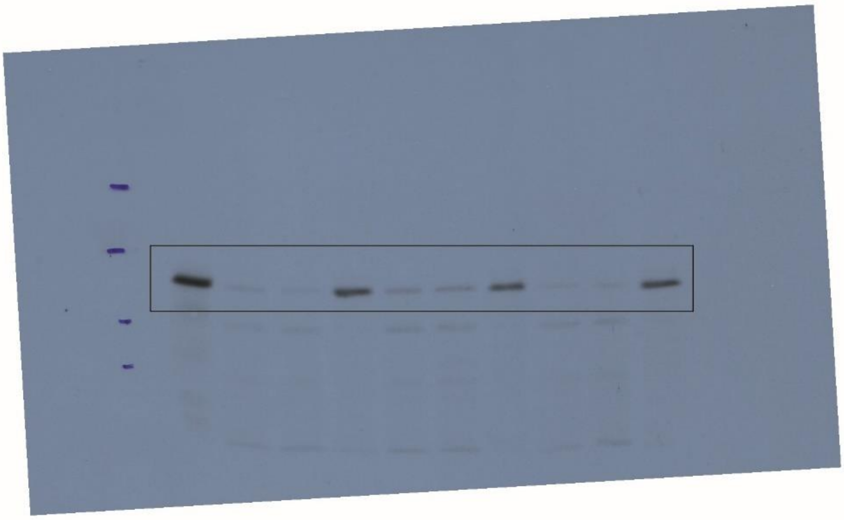

Atp5-DHFR

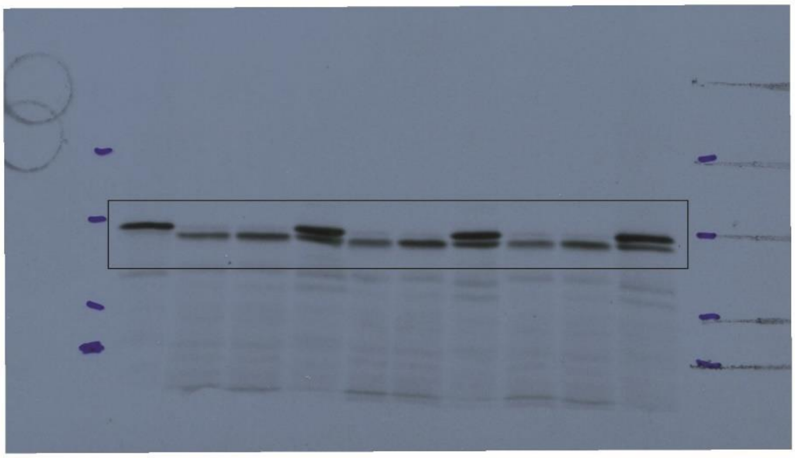

Cor1-DHFR

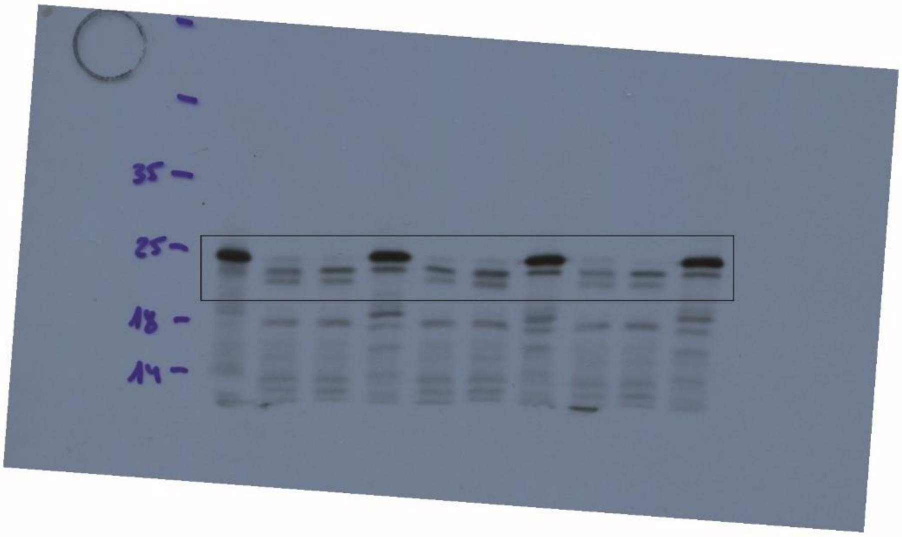

Oxa1-DHFR

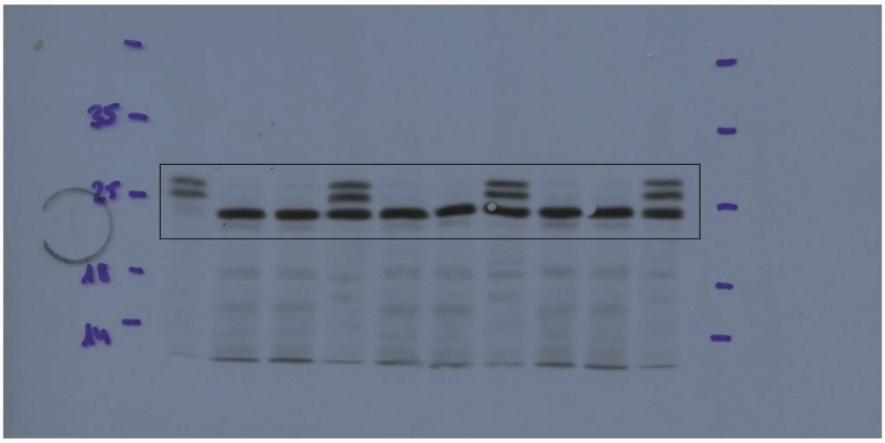

Su9-DHFR

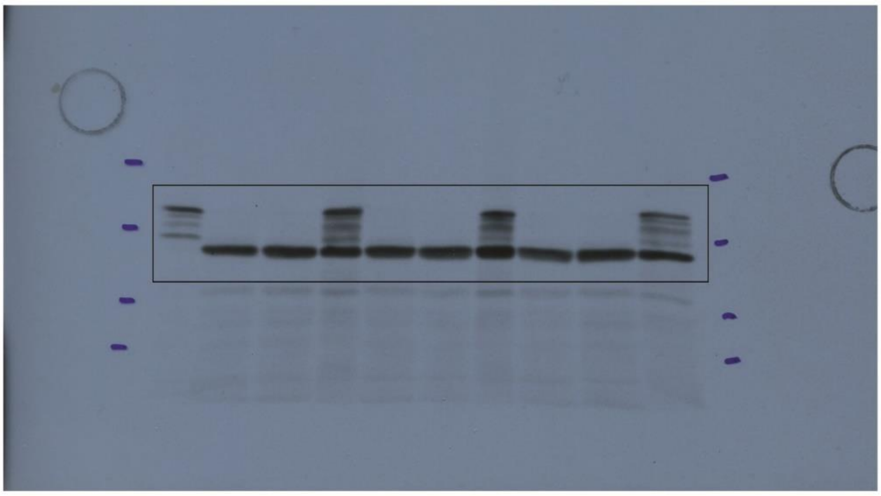

Mdl2-DHFR

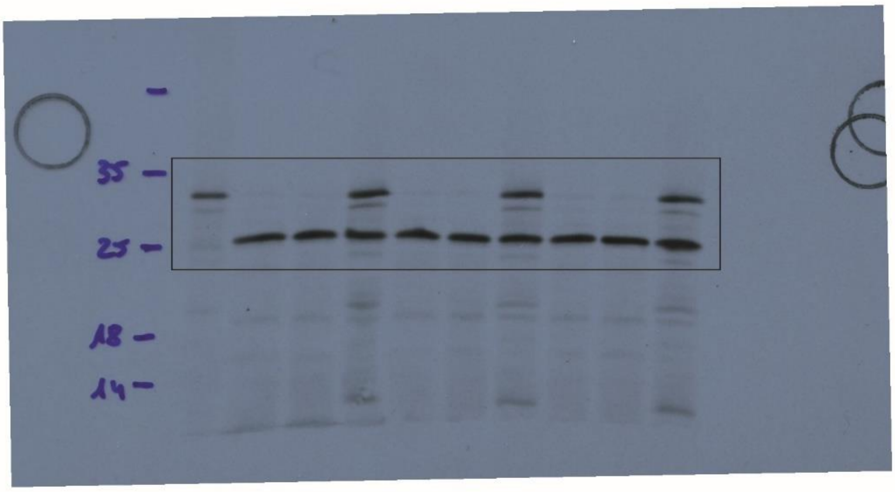

Figure 2D

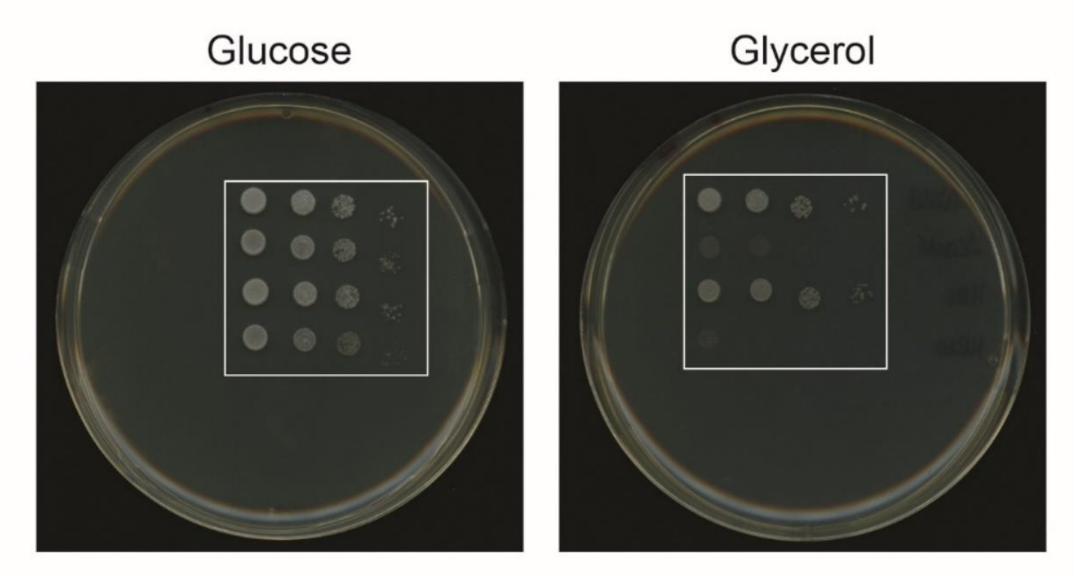

Figure 2E

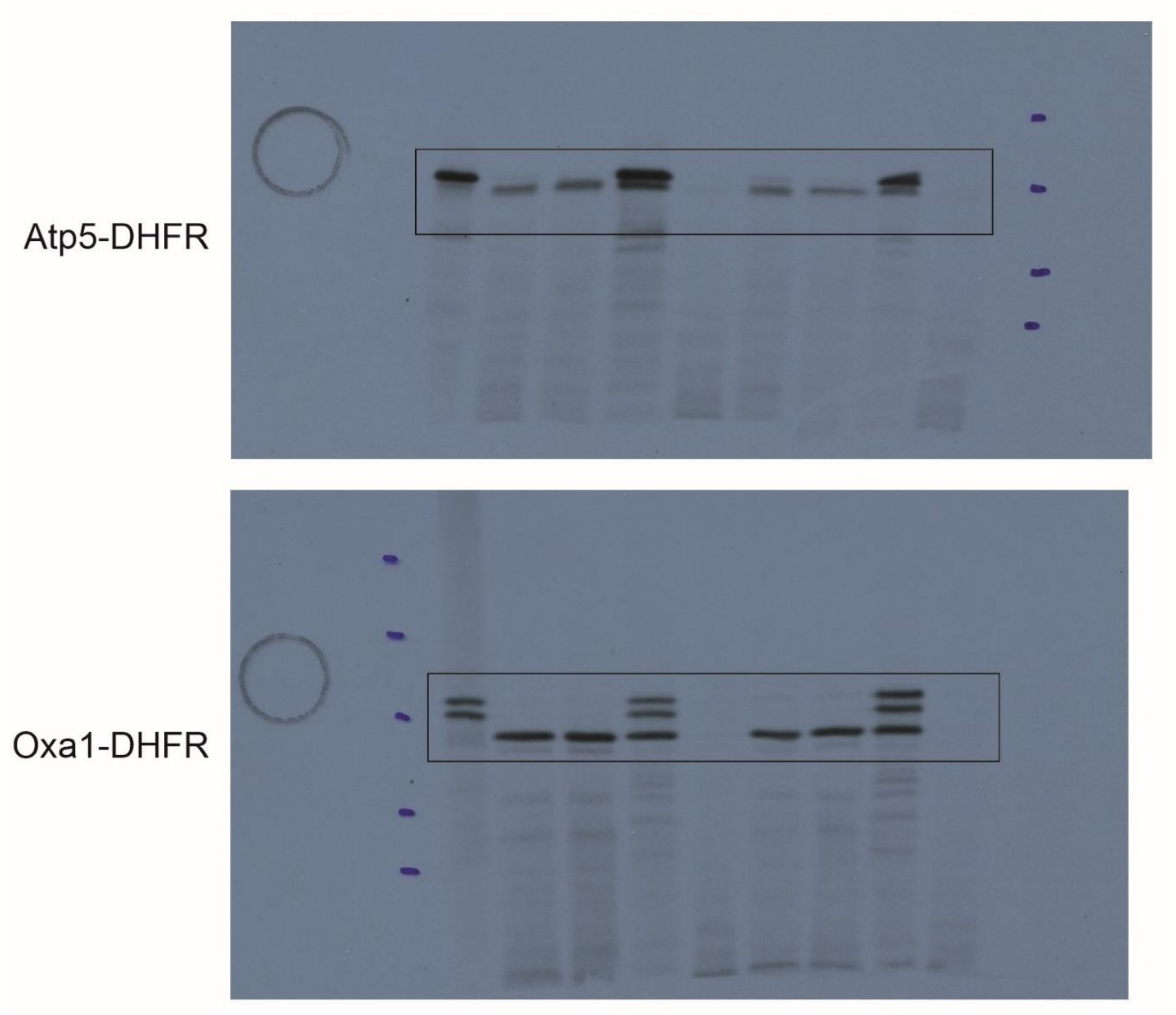

Figure 2F

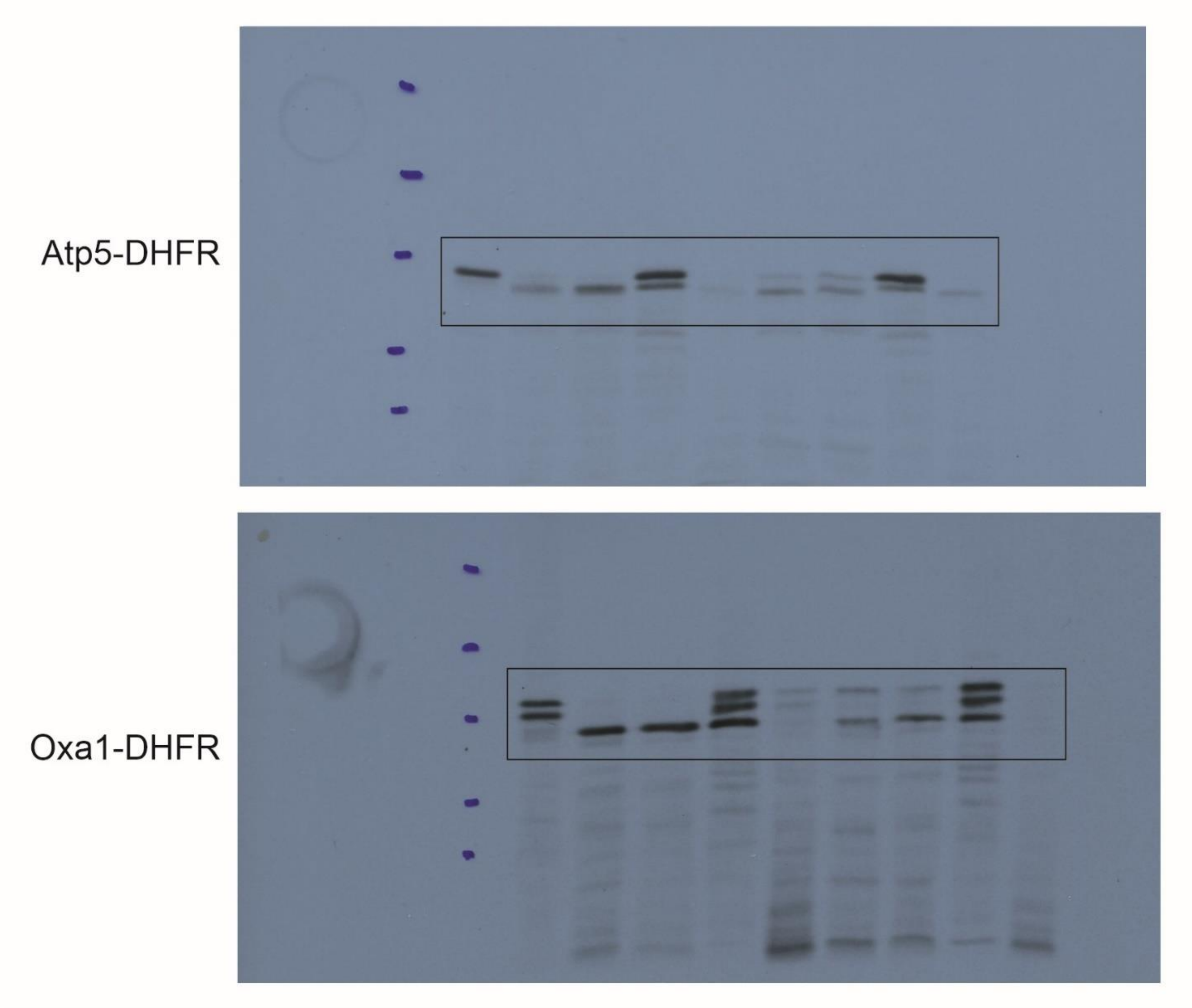

Figure 3

Figure 3B

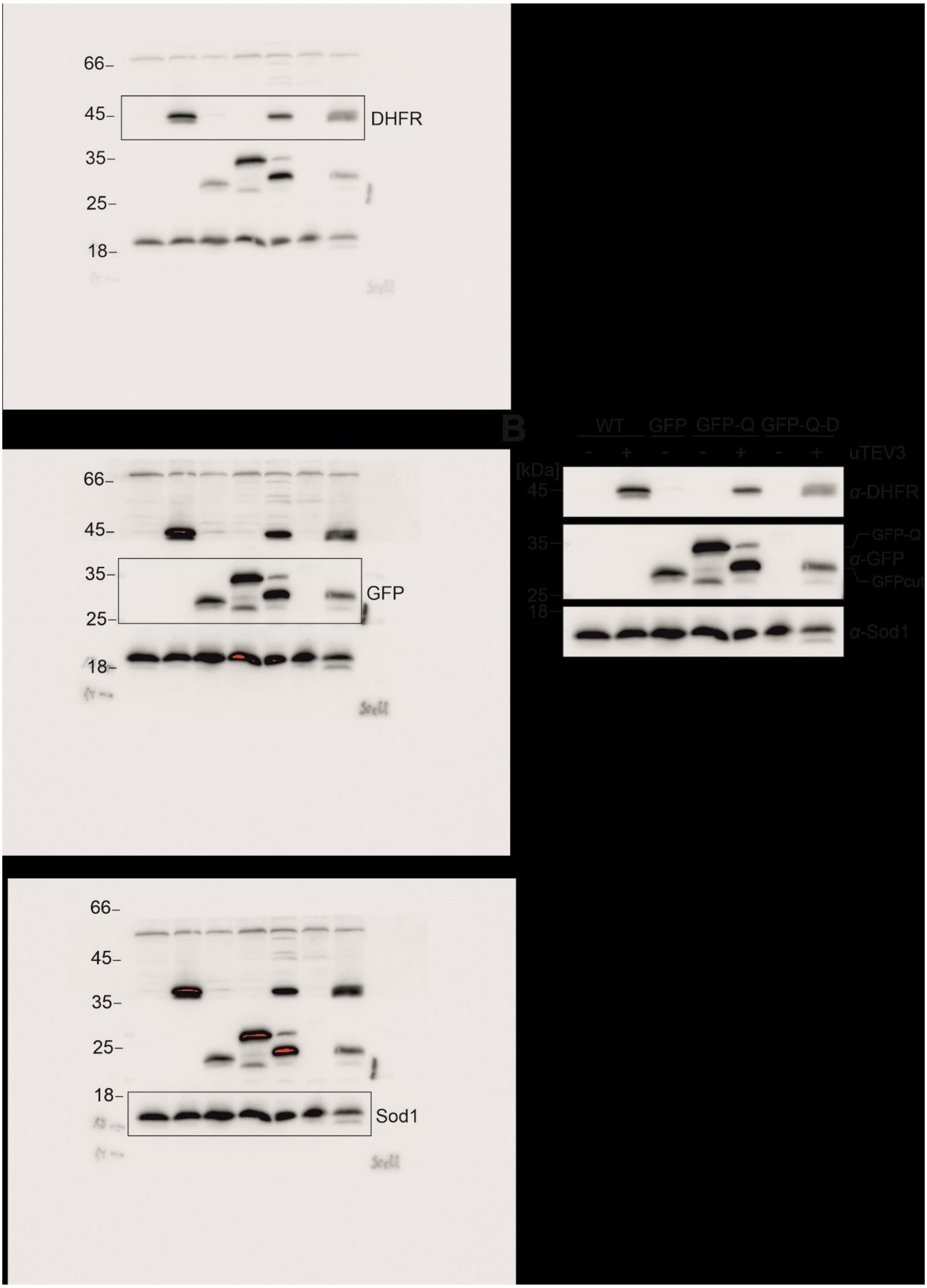

Figure 3D

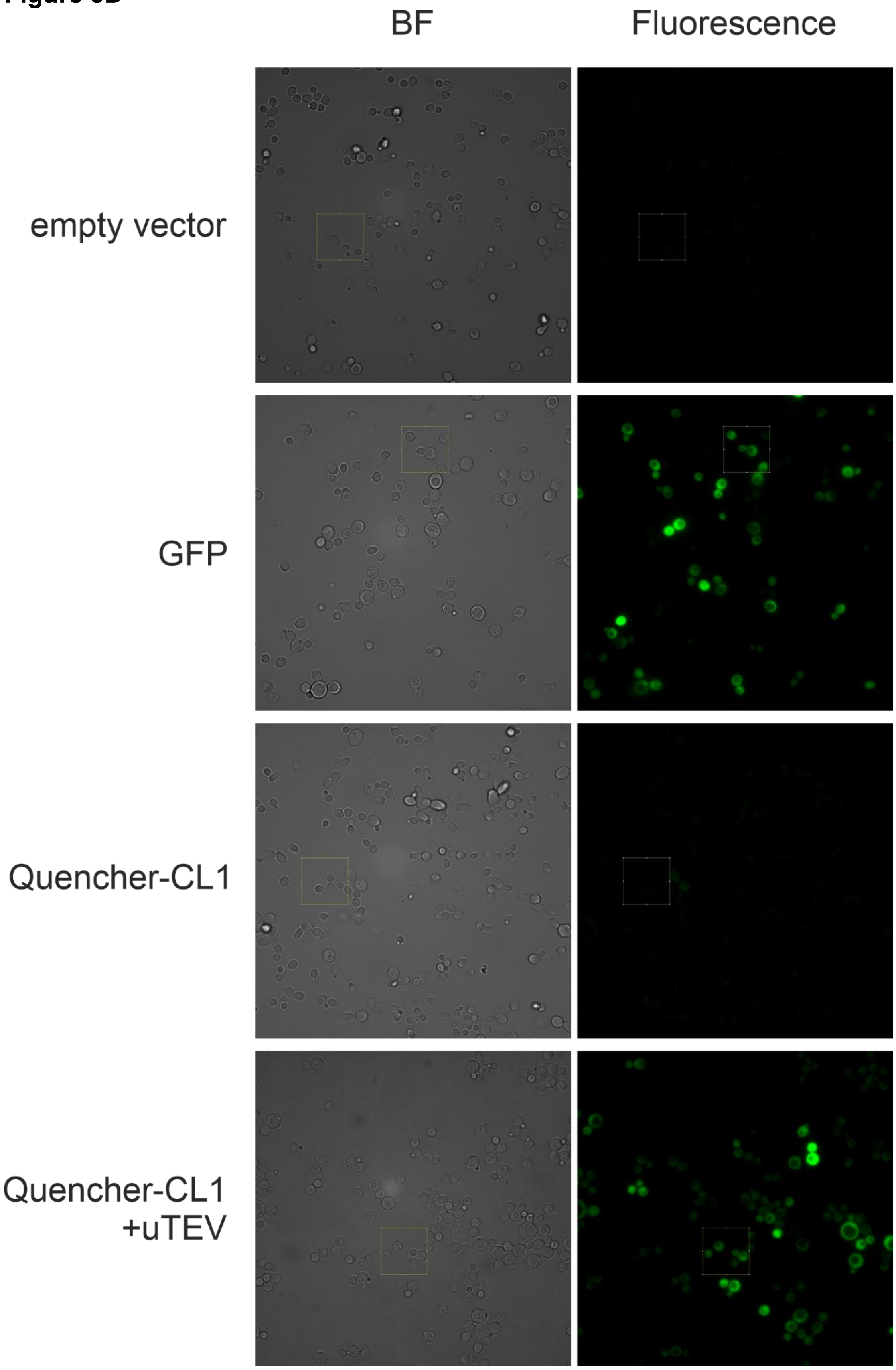
BF
Fluorescence
empty vector
GFP
Quencher-CL1
Quencher-CL1
+uTEV

Figure 4

Figure 4B

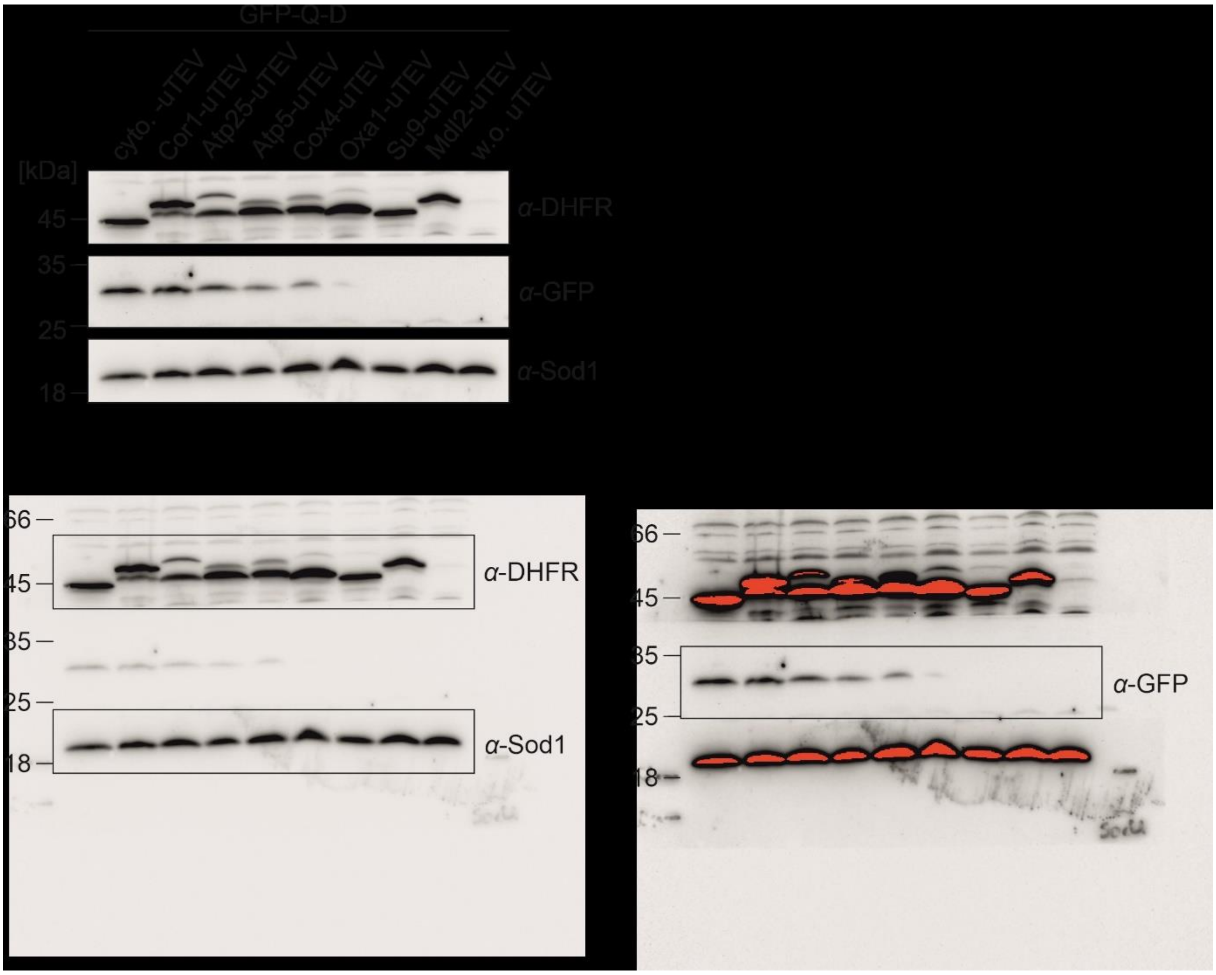

Figure 5

Figure 5A

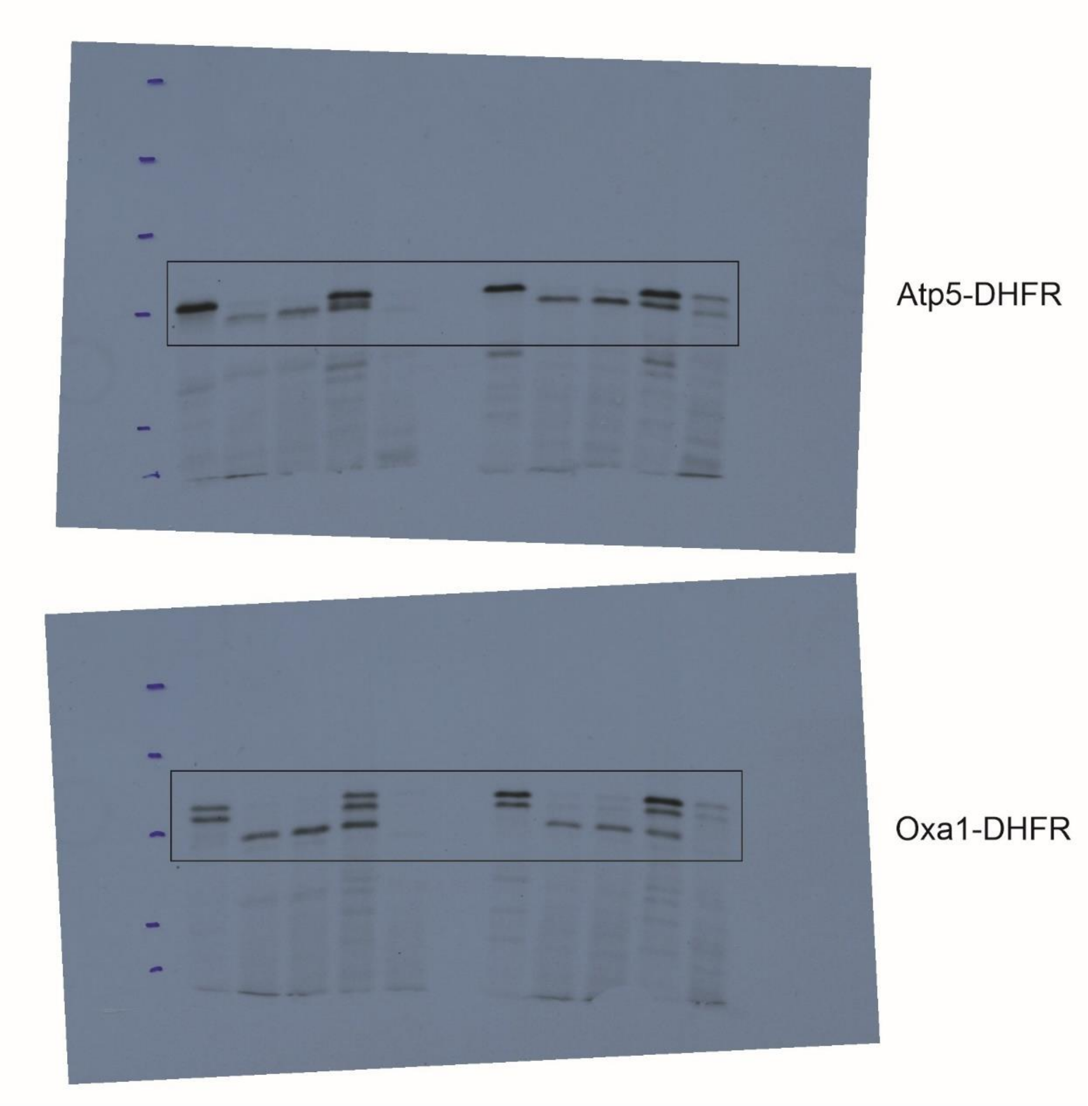

Figure 5B

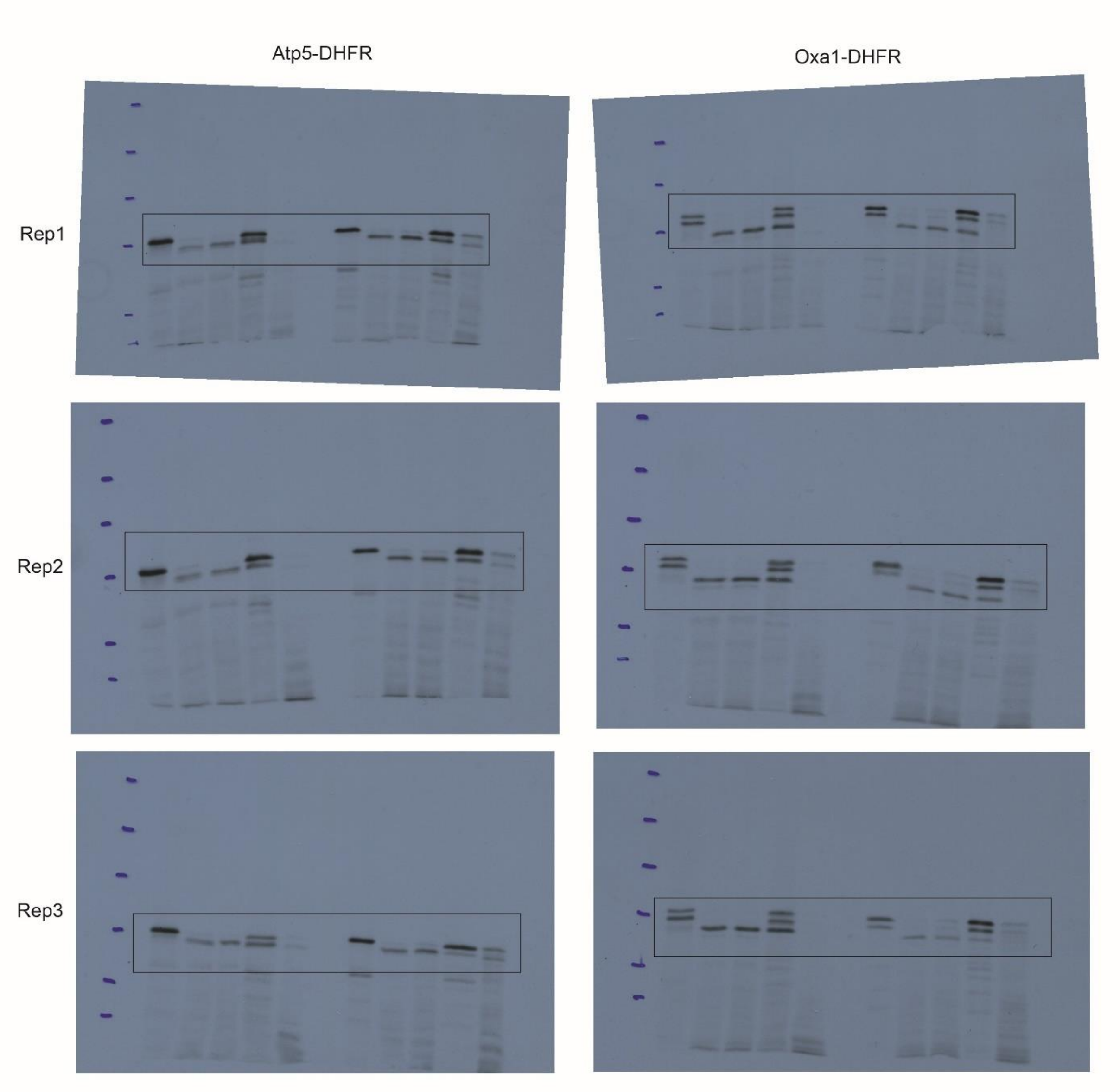

Figure 5C

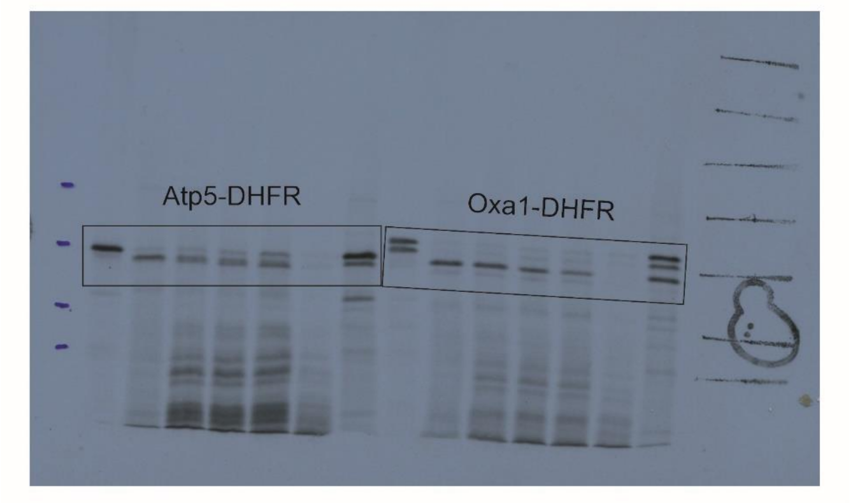

Figure 5D

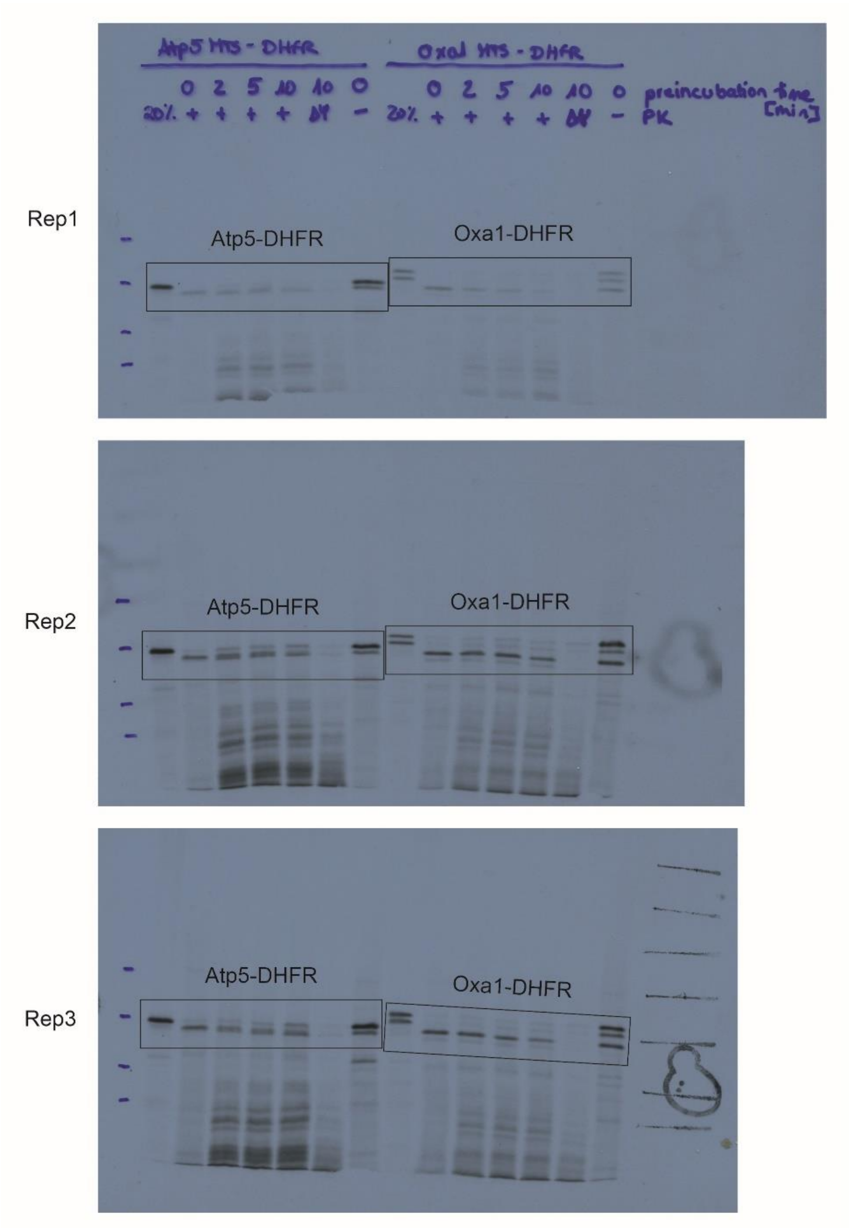
Atp5 MTS - DHFR
Oxa1 MTS - DHFR
0 2 5 10 10 0
0 2 5 10 10 0
preincubation time [min]
20% + + + + ΔΨ -
20% + + + + ΔΨ -
PK
Rep1
Atp5-DHFR
Oxa1-DHFR
Rep2
Atp5-DHFR
Oxa1-DHFR
Rep3
Atp5-DHFR
Oxa1-DHFR

Figure 5E

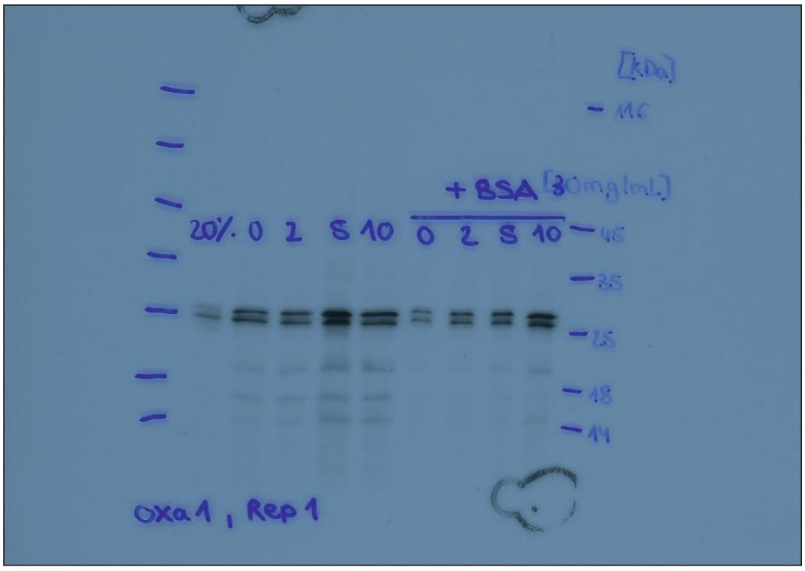
[kDa]
116
+BSA [30mg/mL]
20% 0 2 5 10 0 2 5 10
45
35
25
18
14
Oxa1, Rep 1

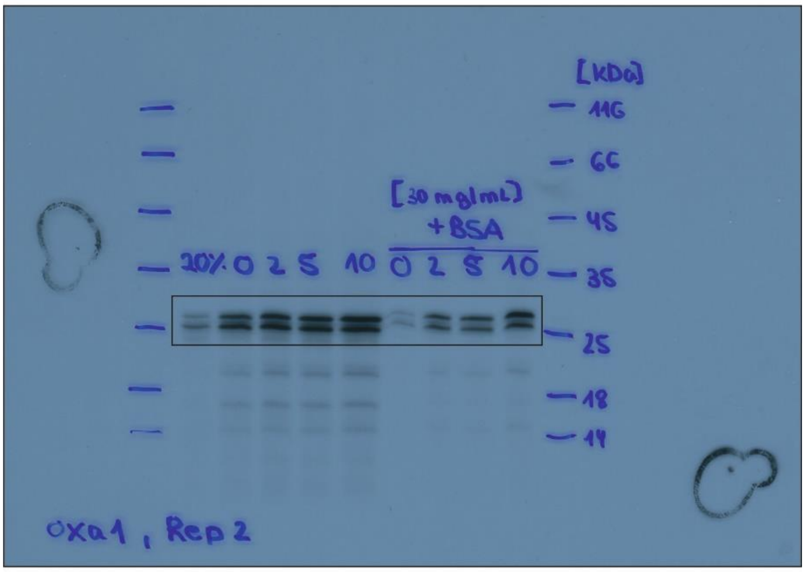
[kDa]
116
66
[30 mg/mL]
+BSA
45
20% 0 2 5 10 0 2 5 10
35
25
18
14
Oxa1, Rep 2

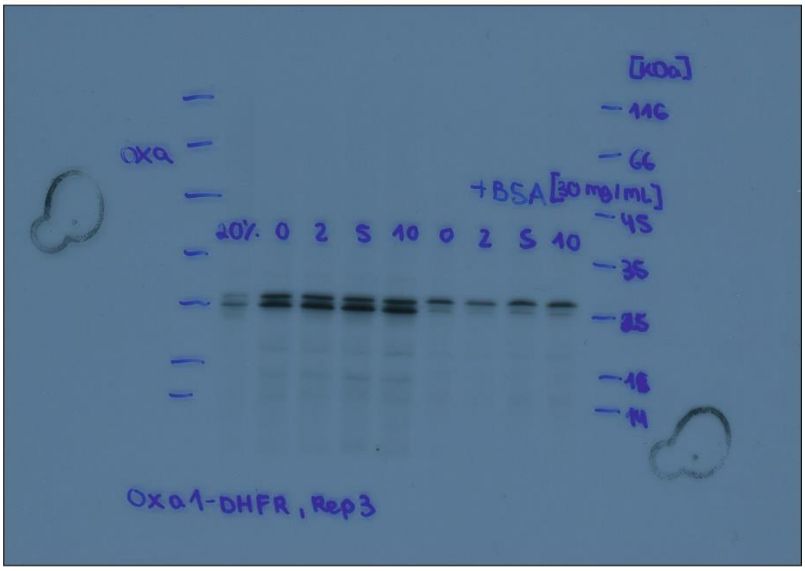
[kDa]
116
Oxa
66
+BSA [30 mg/mL]
45
20% 0 2 5 10 0 2 5 10
35
25
18
14
Oxa1-DHFR, Rep3

Figure 5G

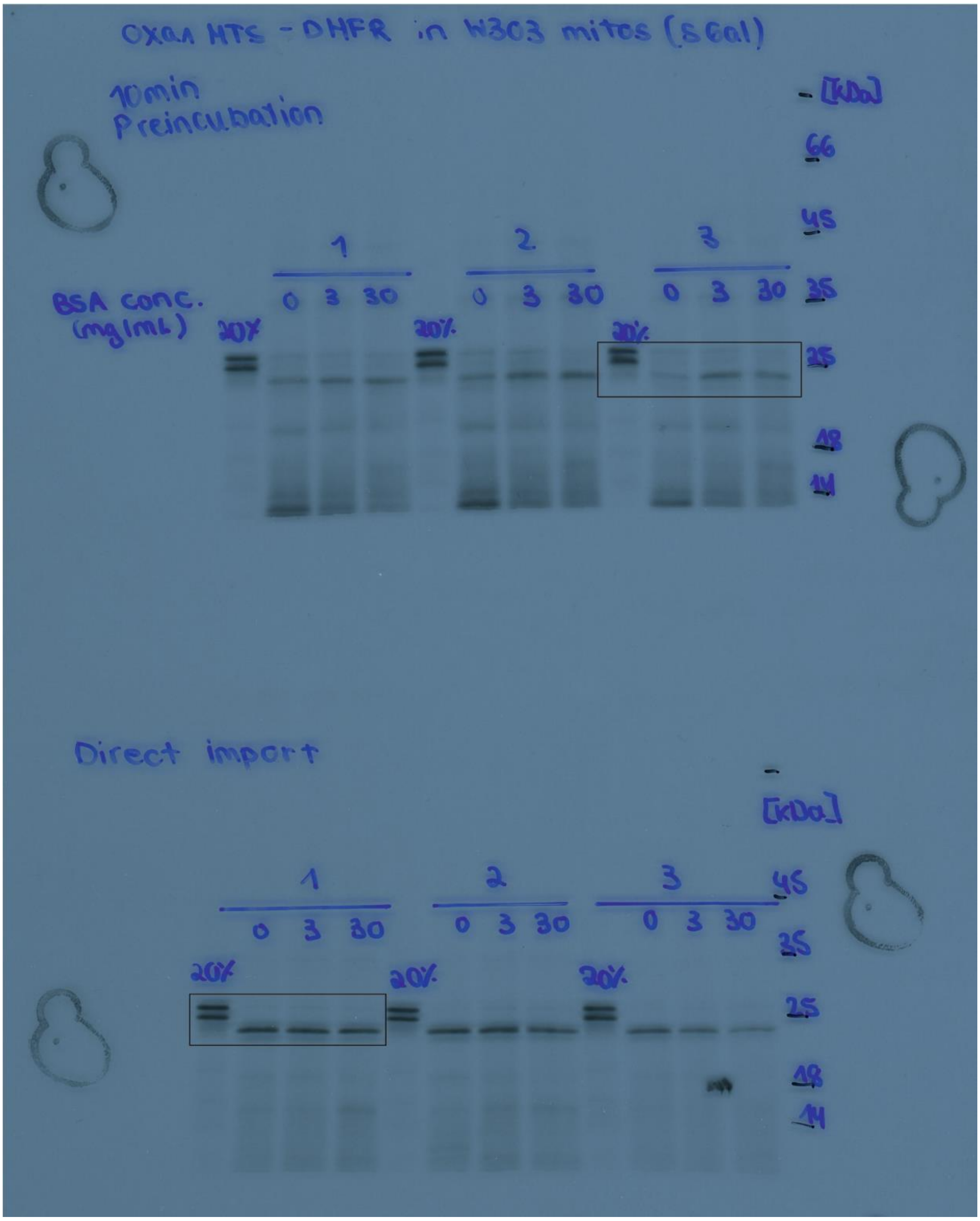
Oxa1 MTS - DHFR in W303 mitos (sGal)
10min Preincubation
[kDa]
66
45
35
25
18
14
1
2
3
BSA conc. (mg/mL)
0 3 30
0 3 30
0 3 30
20%
20%
20%
Direct import
[kDa]
45
35
25
18
14
1
2
3
0 3 30
0 3 30
0 3 30
20%
20%
20%

Figure 5I

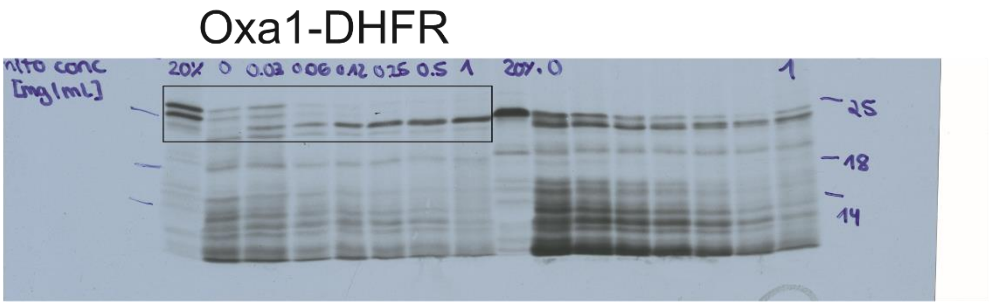

Figure FJ

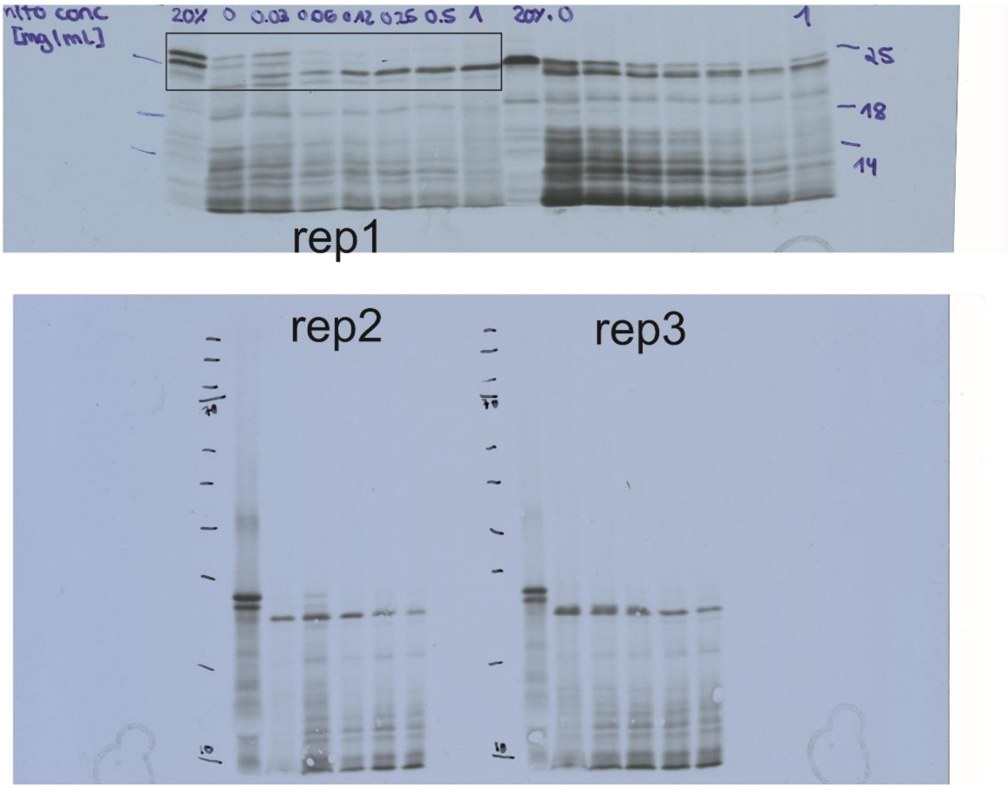

Figure 5K

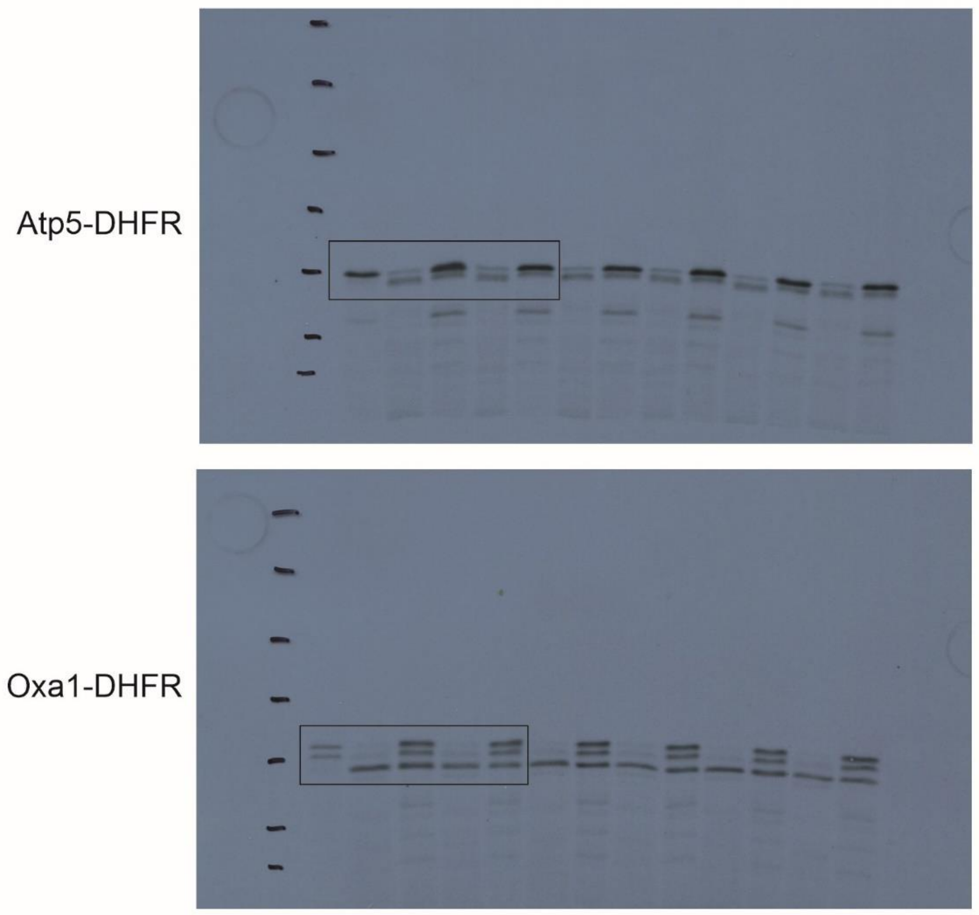
Atp5-DHFR
Oxa1-DHFR

Figure 5L

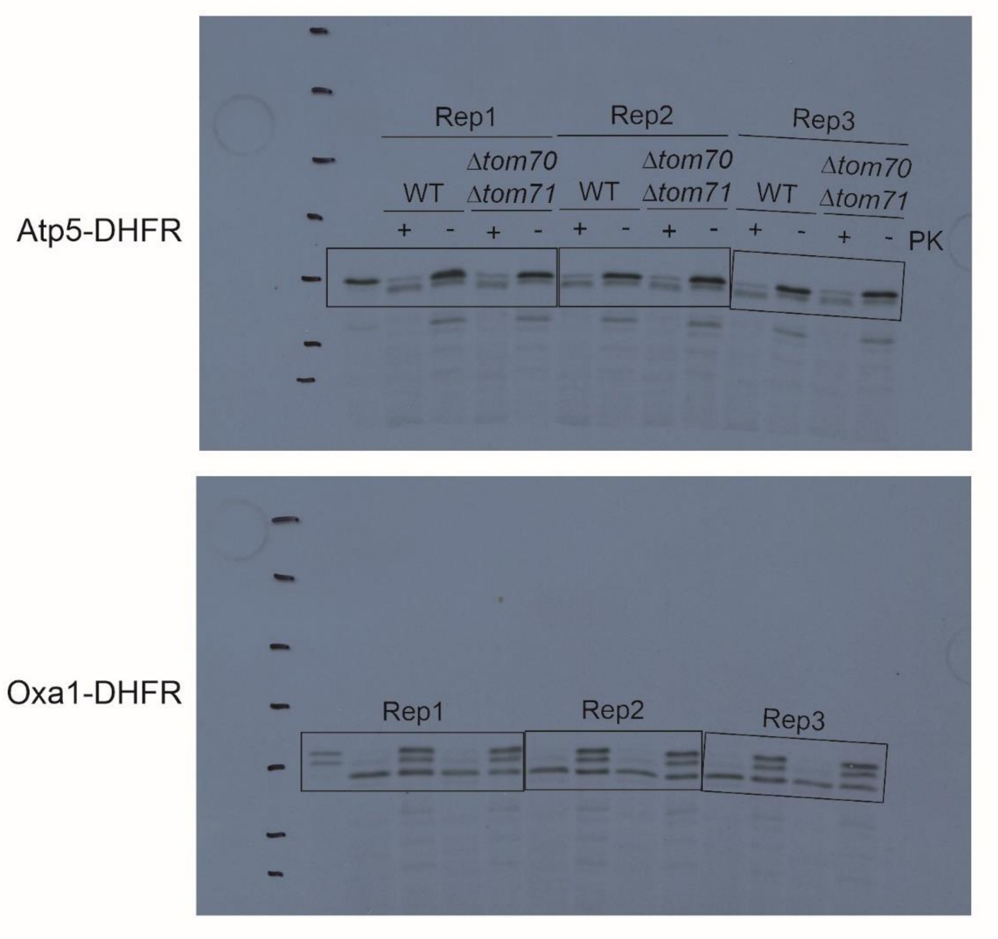

Figure 6

Figure 6F

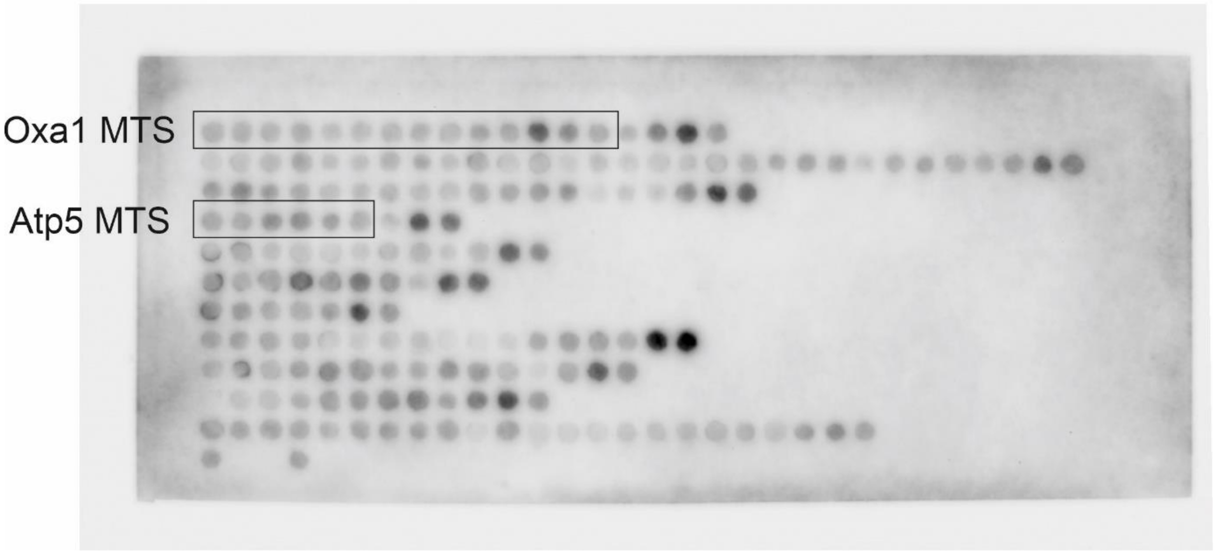

Figure 7

Figure 7A

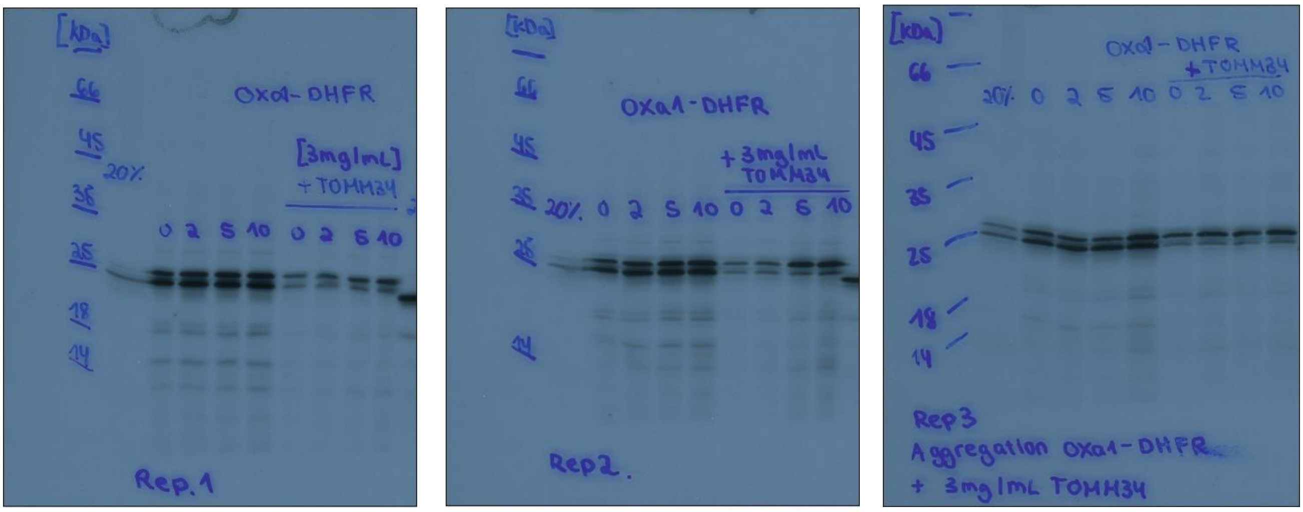

Figure 7D and F

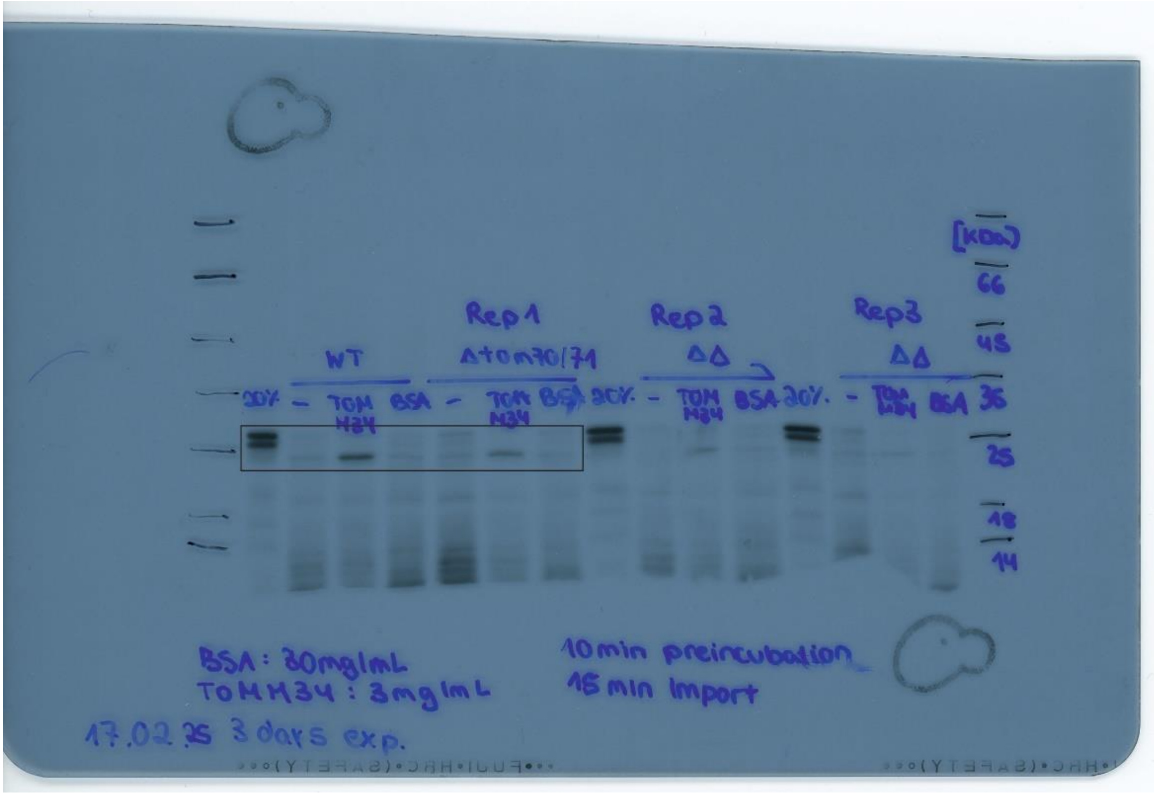

Figure 7E

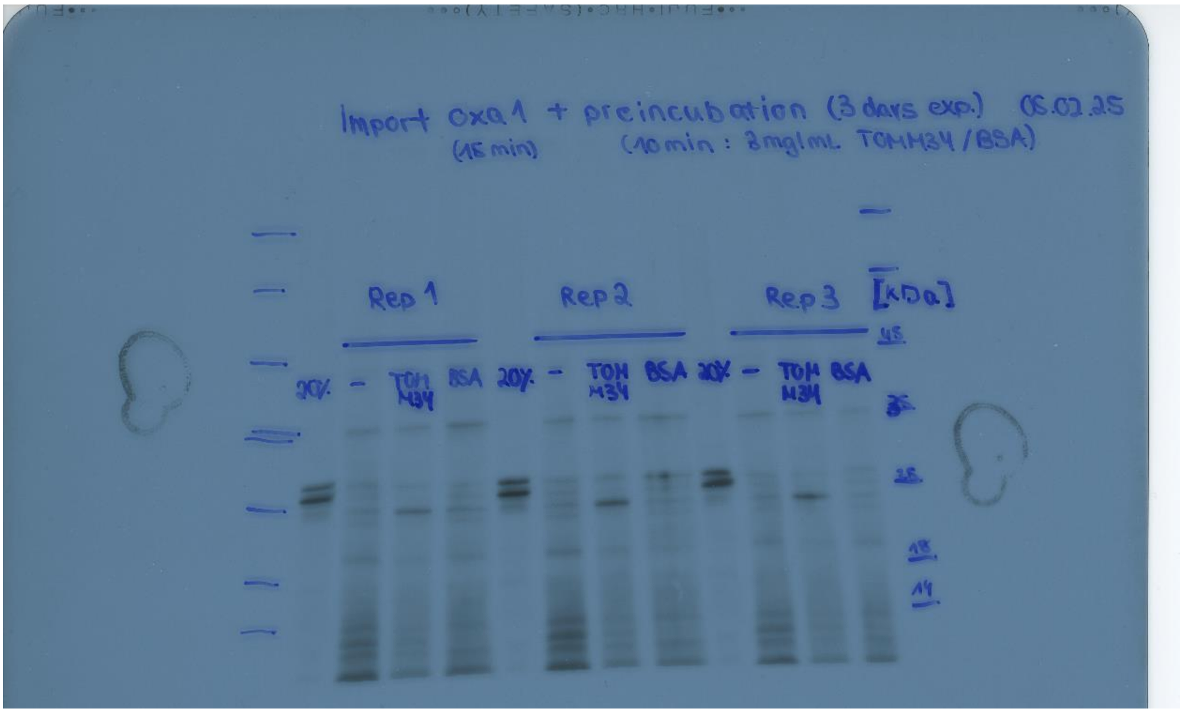

Figure 7I

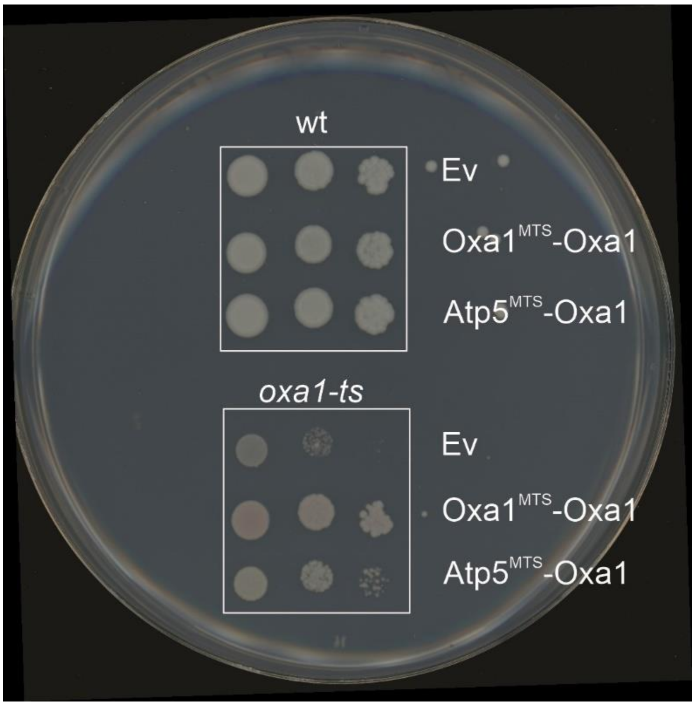

Figure 7G

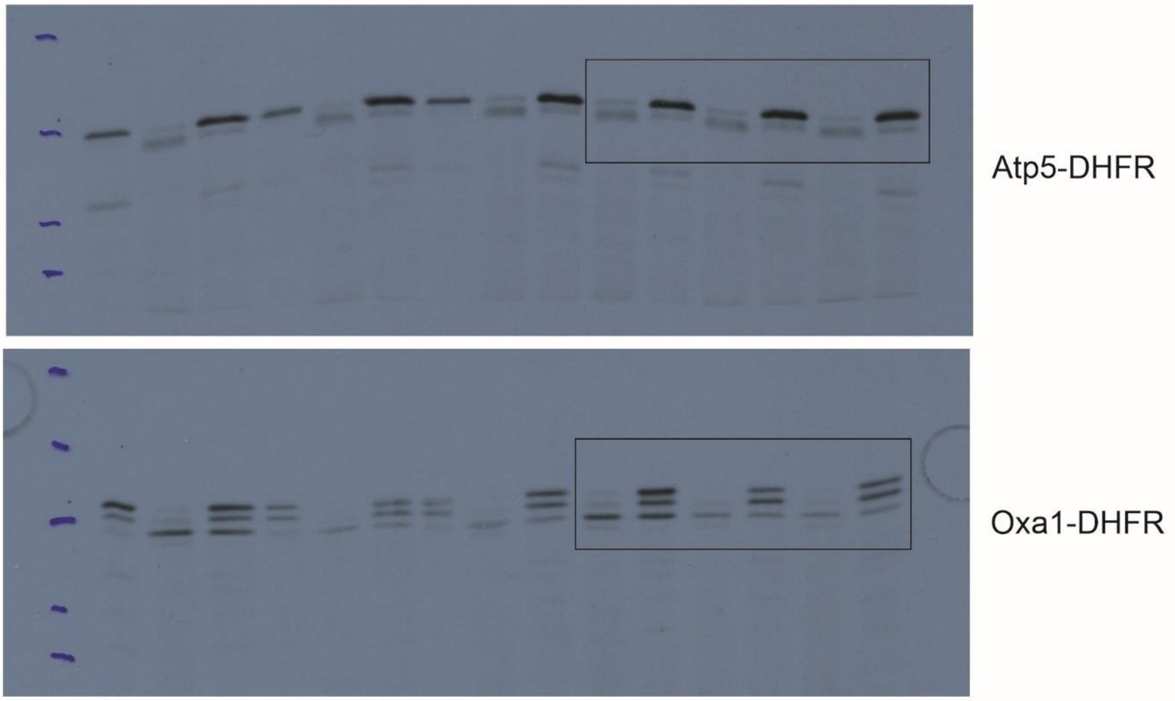

Figure 7H

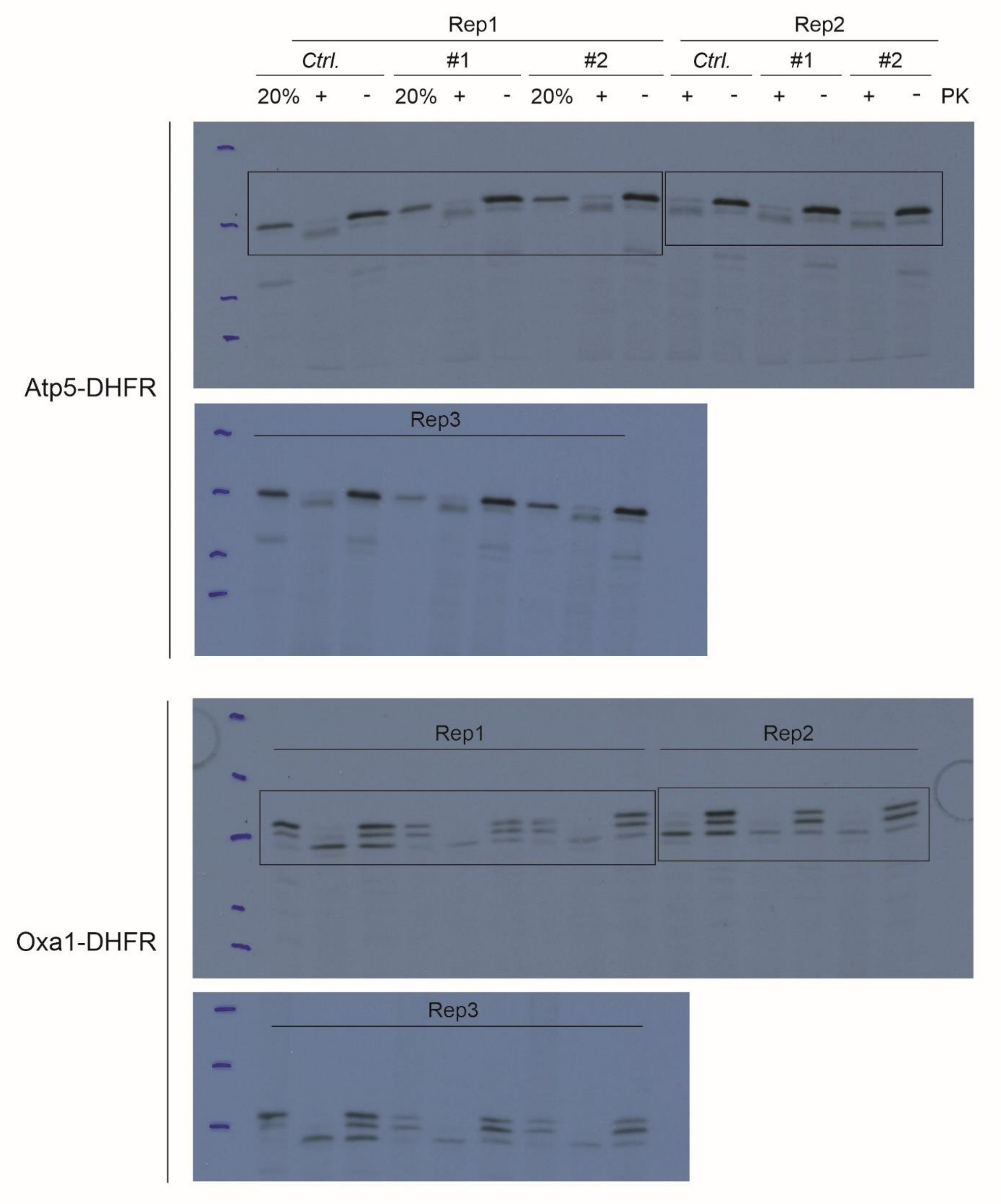

Rep1
Rep2
Ctrl.
#1
#2
Ctrl.
#1
#2
20% + - 20% + - 20% + - + - + - + - PK
Atp5-DHFR
Rep3
Oxa1-DHFR
Rep1
Rep2
Rep3

Figure 8

Figure 8B

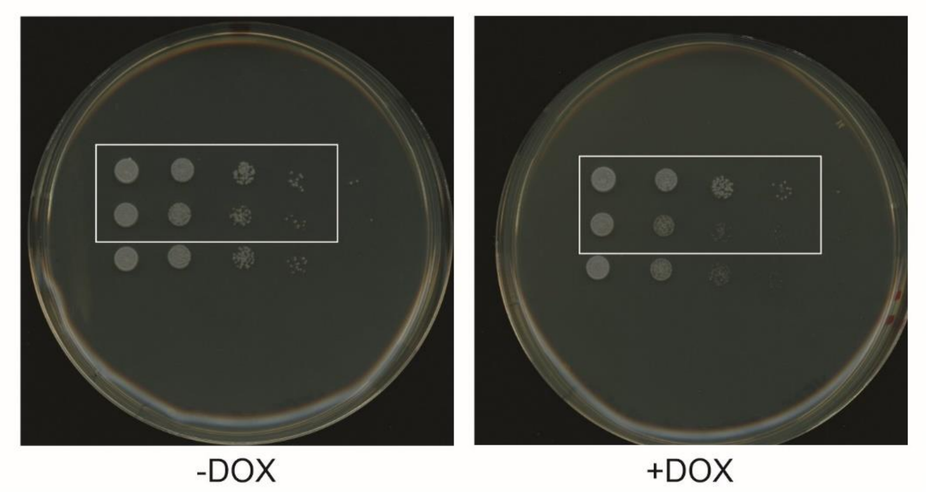

Figure 8C

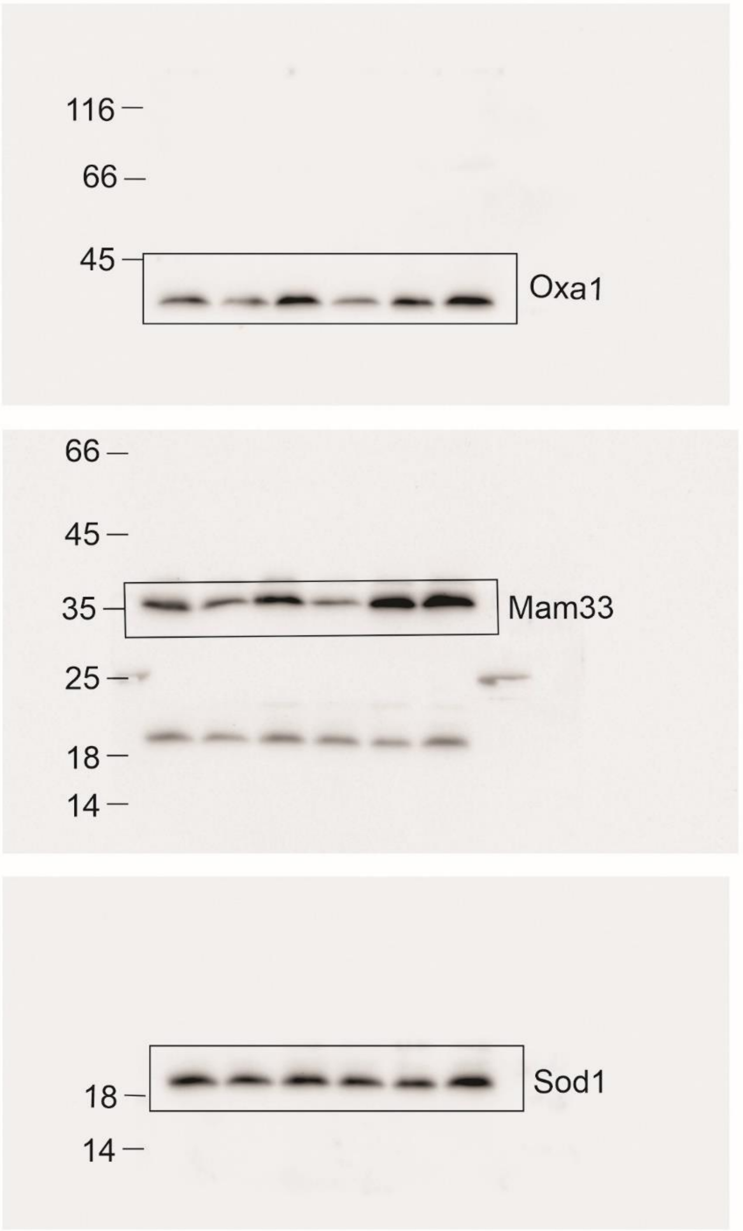

Figure 8D

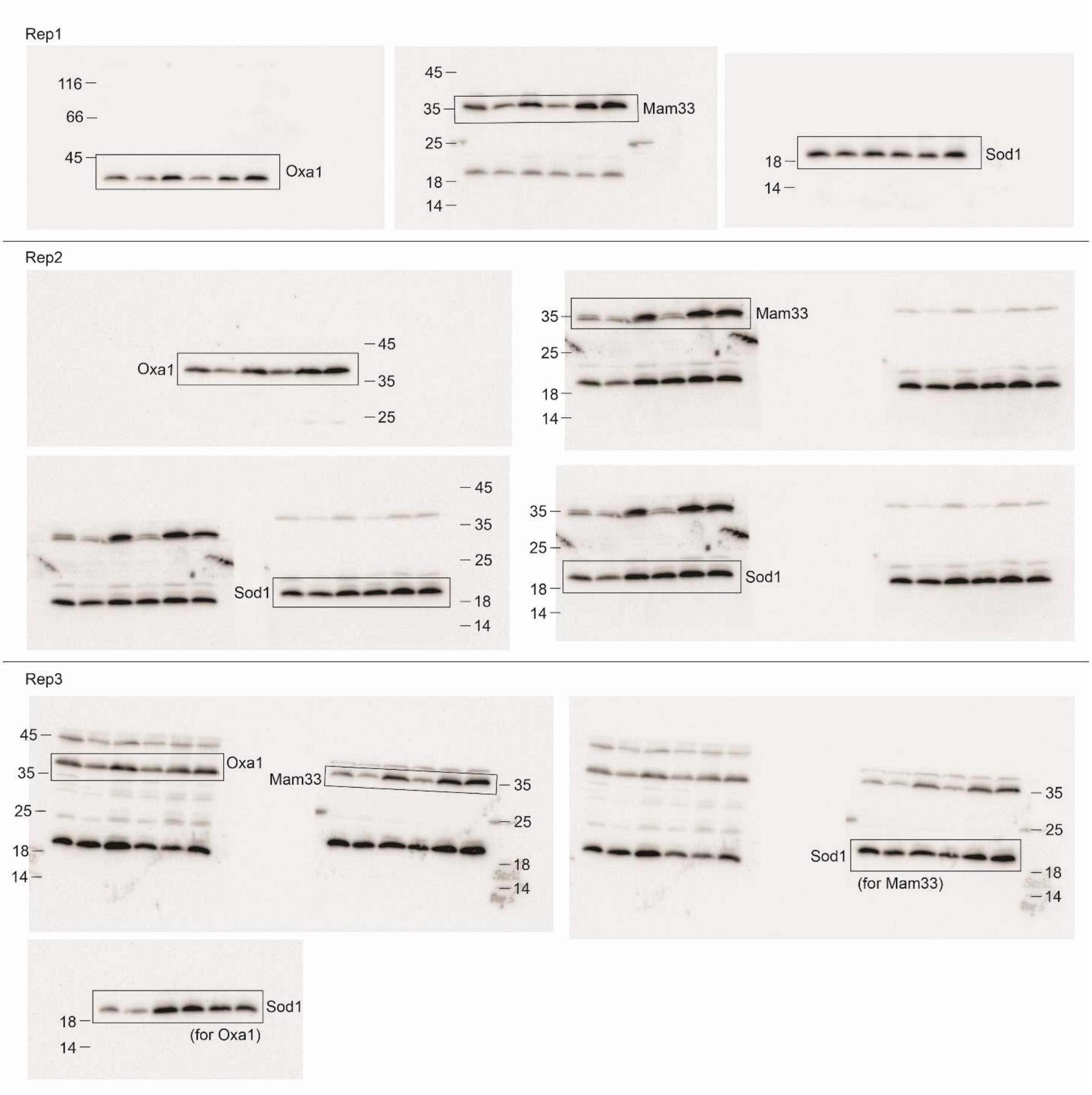
Rep1
116
66
45
Oxa1
45
35
Mam33
25
18
14
18
Sod1
14
Rep2
Oxa1
45
35
25
35
Mam33
25
18
14
45
35
25
Sod1
18
14
35
25
18
Sod1
14
Rep3
45
35
Oxa1
25
18
14
Mam33
35
25
18
14
35
25
Sod1
(for Mam33)
18
14
18
Sod1
(for Oxa1)
14

Figure S3

Figure S3B

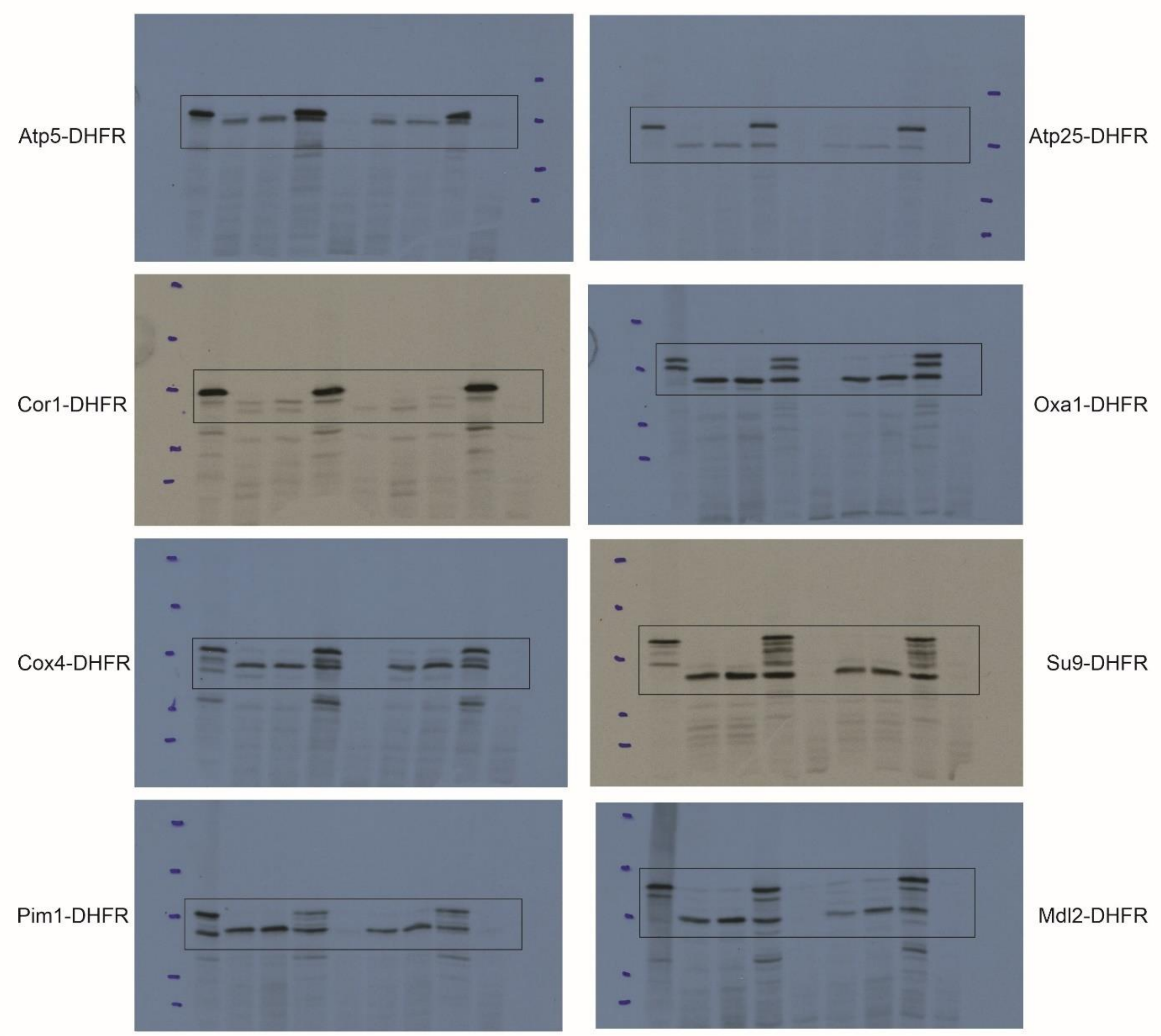

Figure S3C

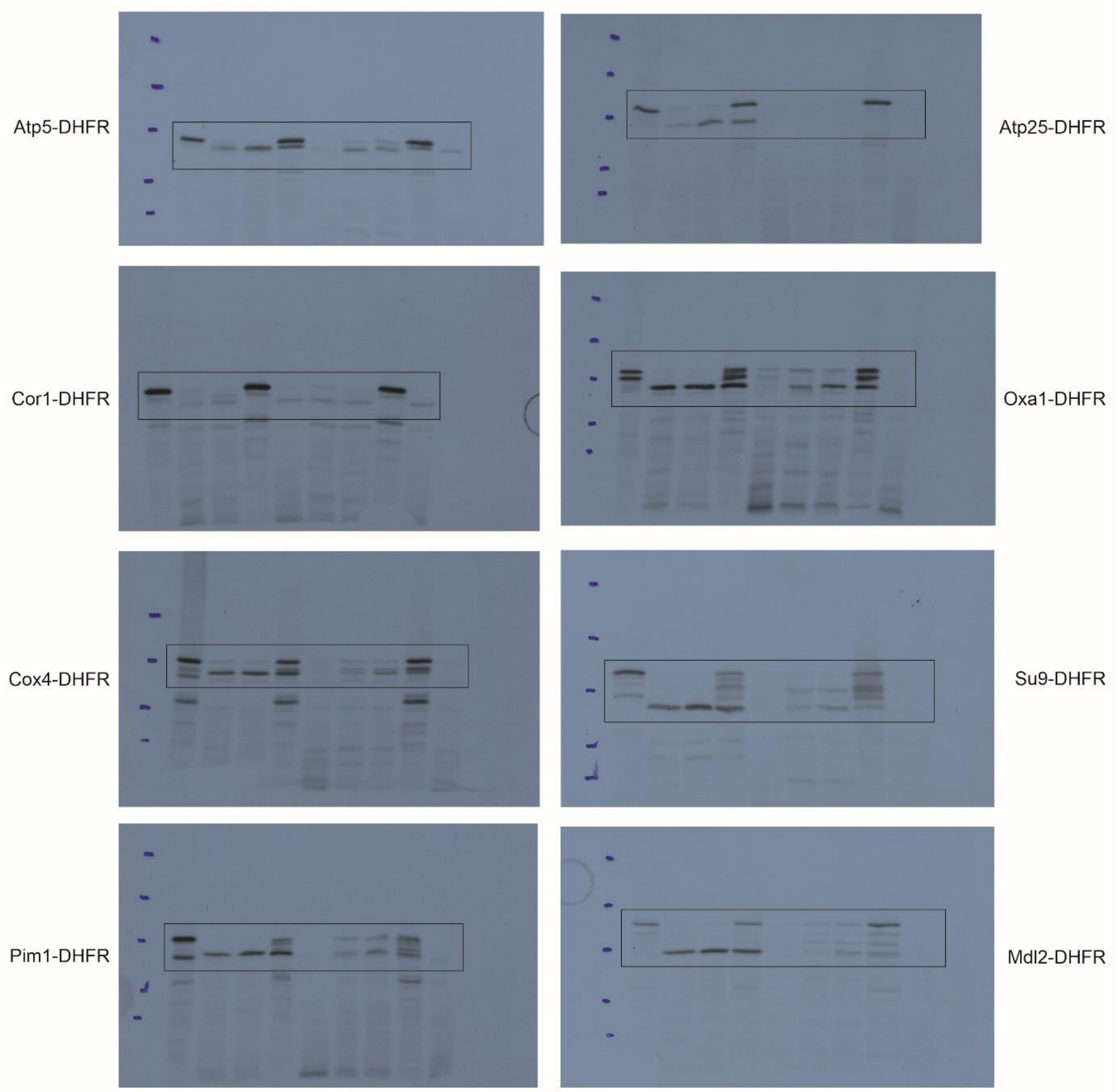
Atp5-DHFR
Atp25-DHFR
Cor1-DHFR
Oxa1-DHFR
Cox4-DHFR
Su9-DHFR
Pim1-DHFR
Mdl2-DHFR

Figure S4

Figure S4E

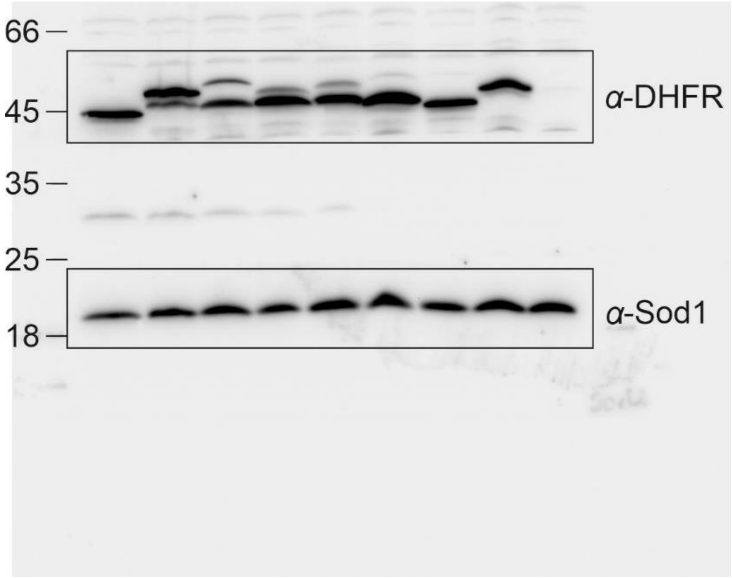

Figure S4F

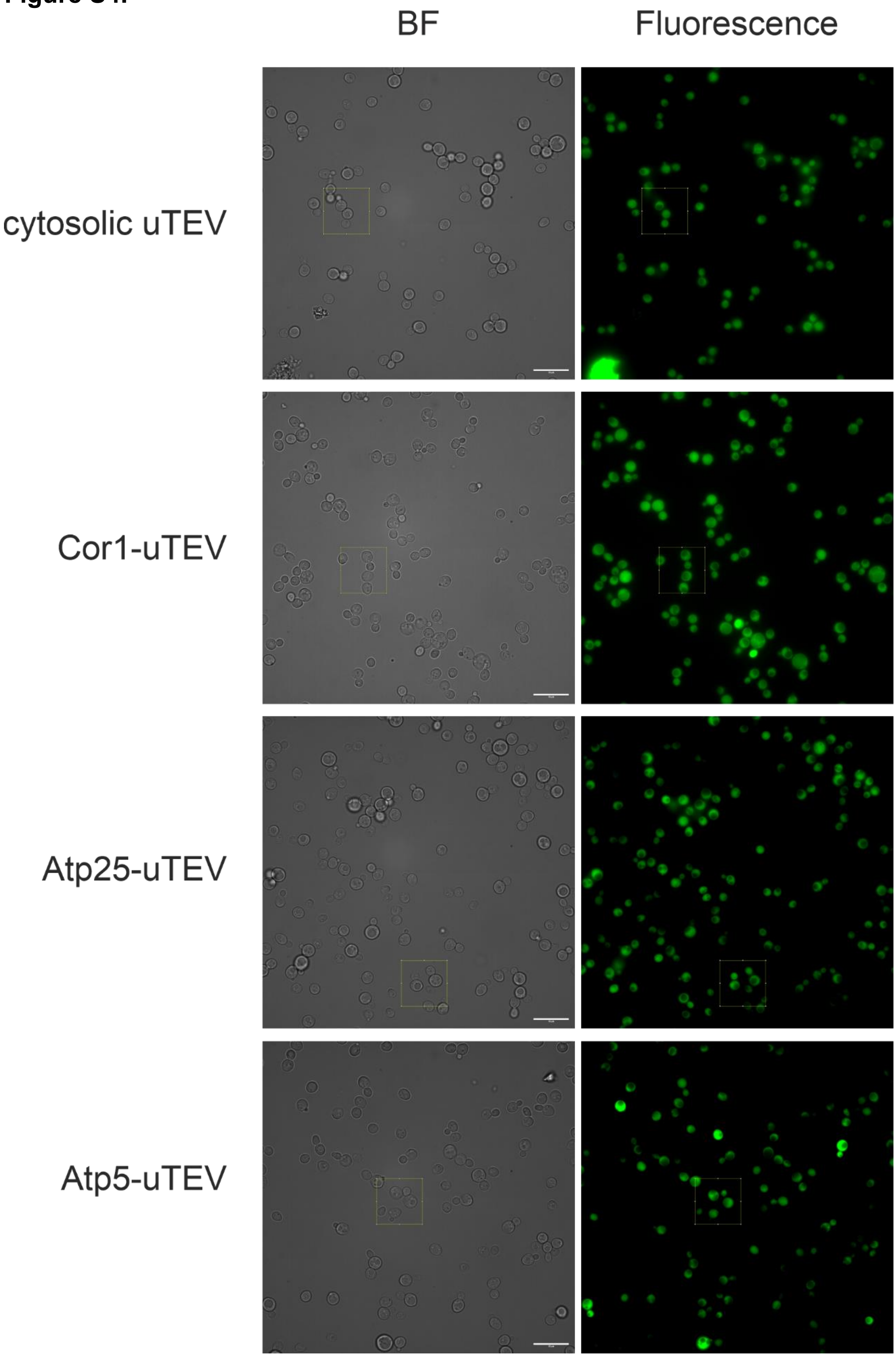
BF
Fluorescence
cytosolic uTEV
Cor1-uTEV
Atp25-uTEV
Atp5-uTEV

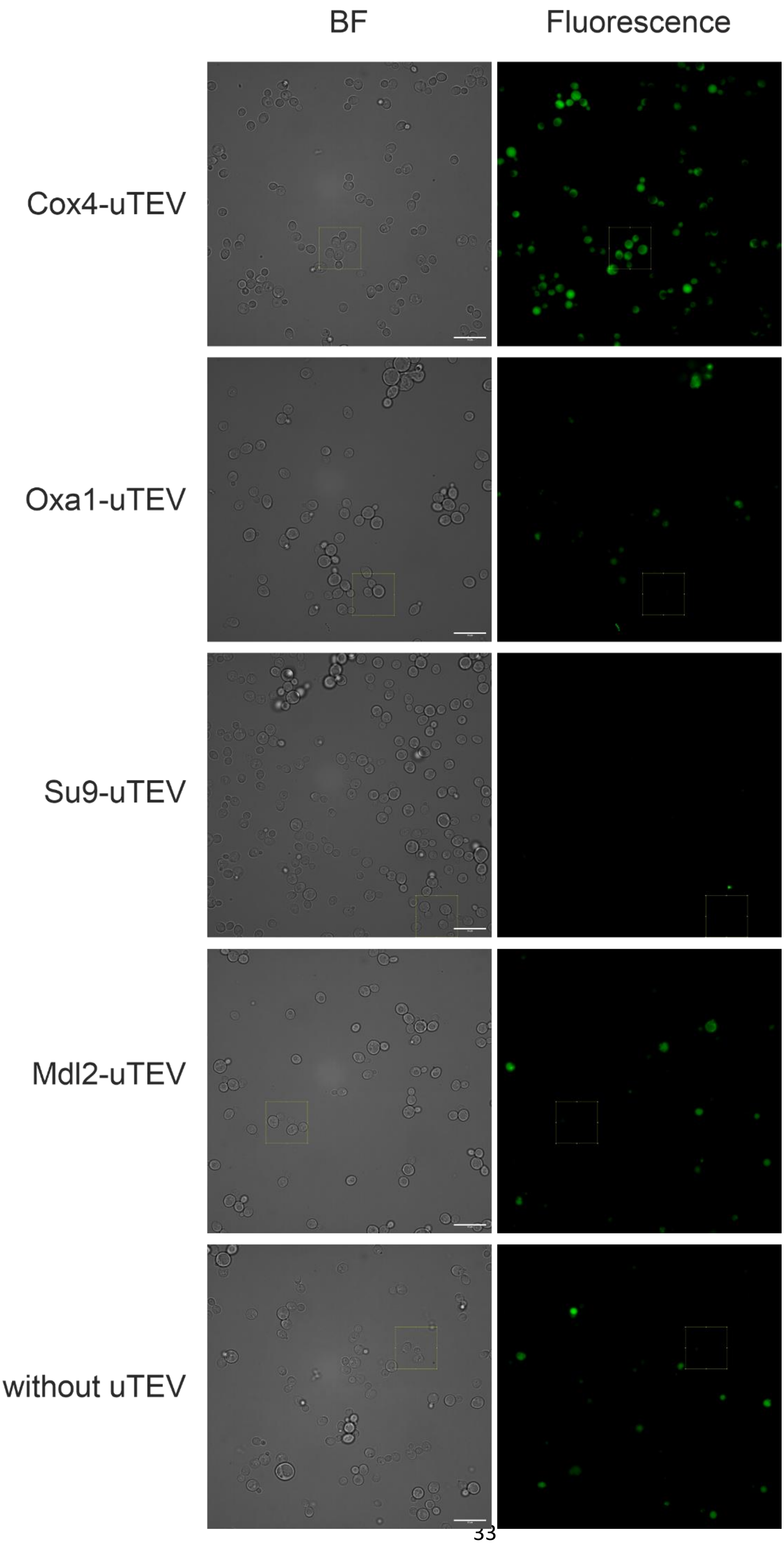

BF
Fluorescence
Cox4-uTEV
Oxa1-uTEV
Su9-uTEV
Mdl2-uTEV
without uTEV

Figure S5

Figure S5A

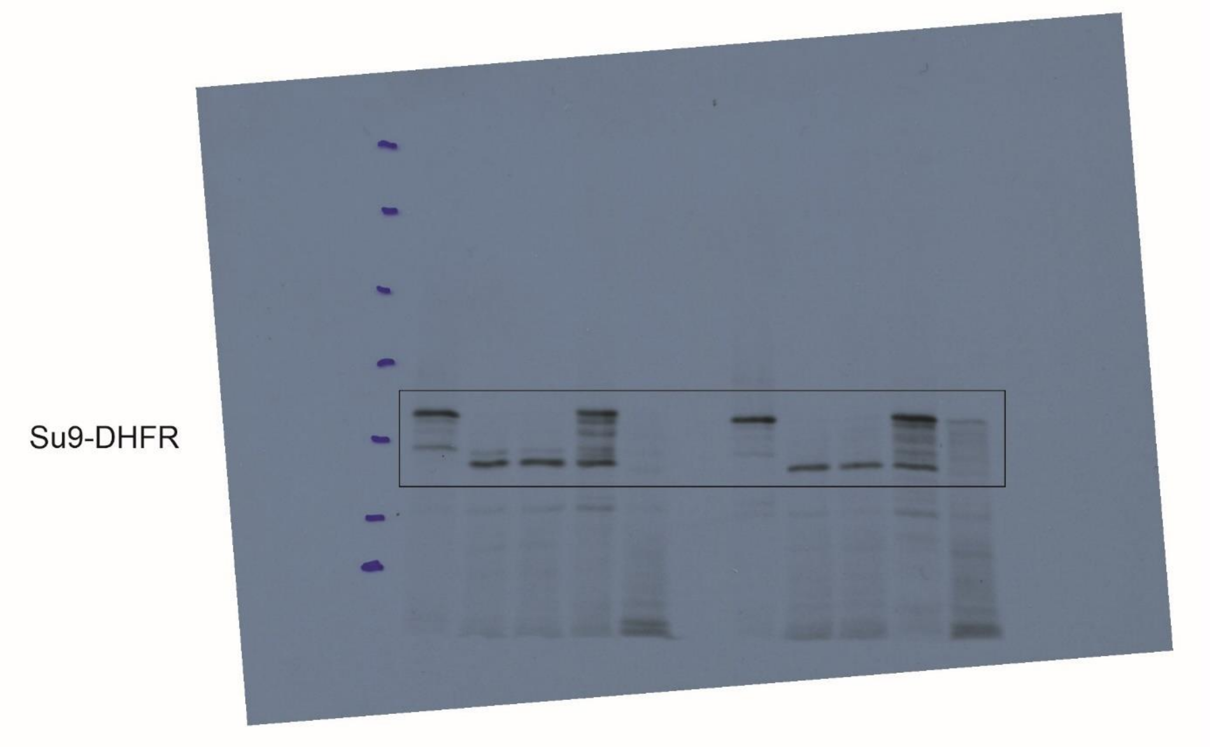

Figure S5B

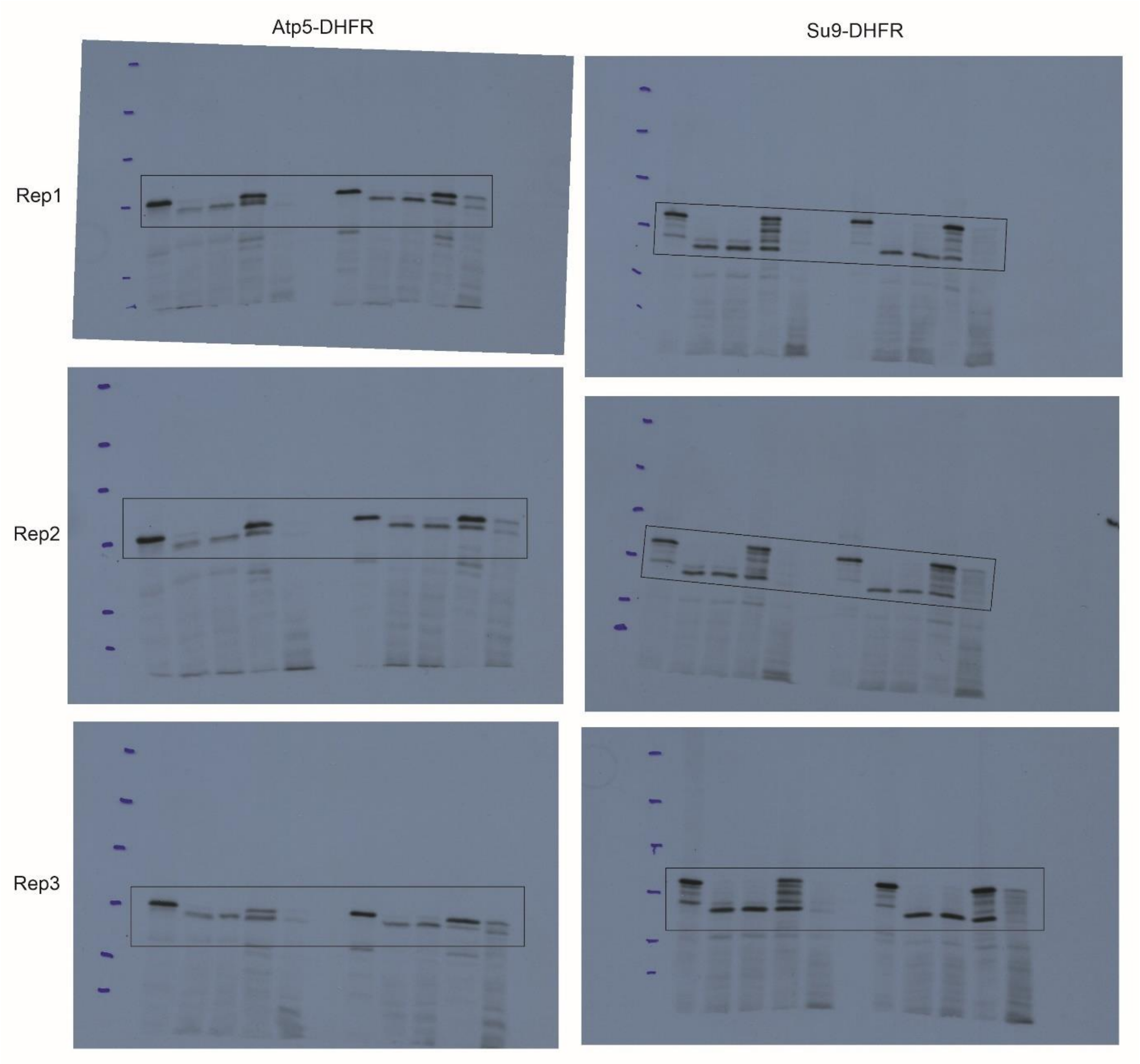

Figure S5E

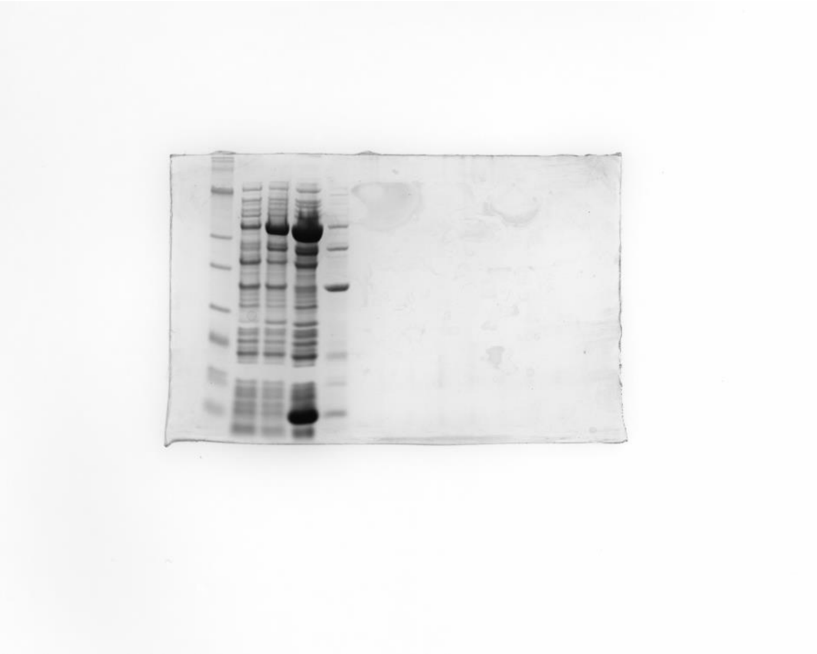

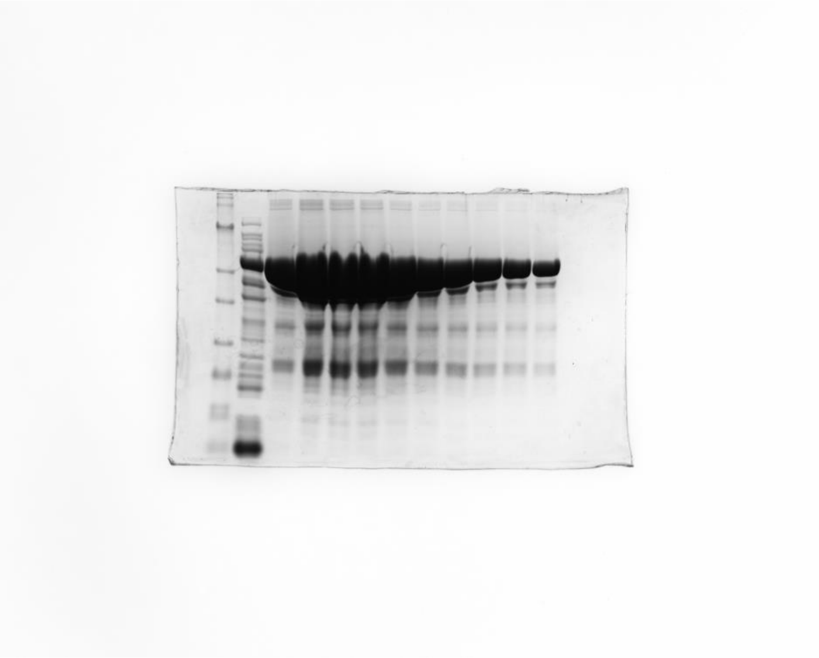

Figure S6

Figure S6E

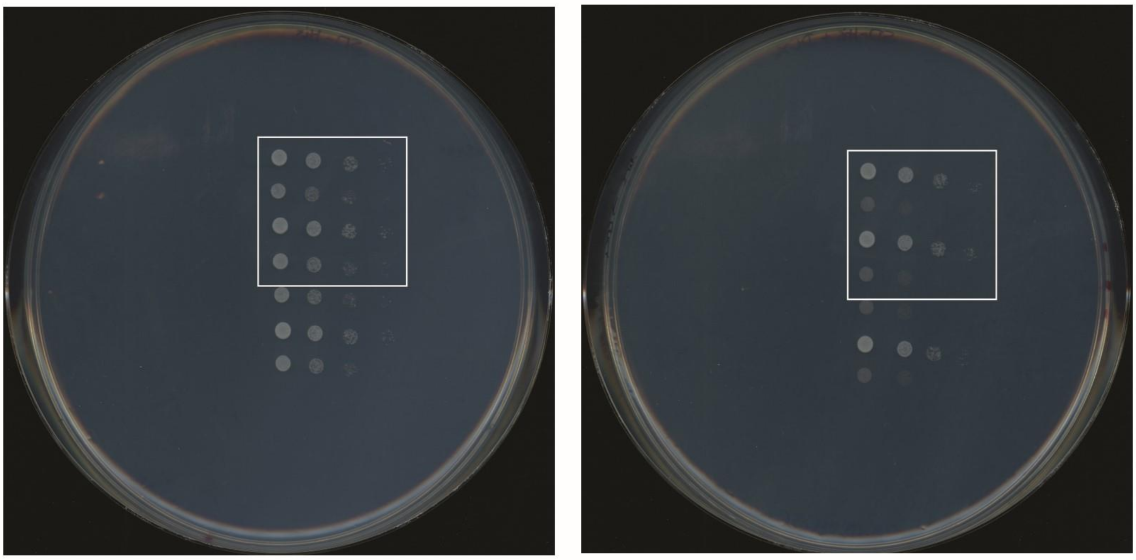

-DOX +DOX
