# Supplementary material for: A protein-specific priority code in presequences determines the efficiency of mitochondrial protein import
Source: PLoS Biol. 2025 Jul 21;23(7):e3003298. doi: 10.1371/journal.pbio.3003298 (PMC12306757; doi:10.1371/journal.pbio.3003298)
Supplement: S1 Table — Refers to S1B Fig. (PDF) [file pbio.3003298.s009.pdf]

Organism	counts
Homo sapiens (Human)	472
Mus musculus (Mouse)	232
Arabidopsis thaliana (Mouse-ear cress)	181
Saccharomyces cerevisiae (strain ATCC 204508 / S288c) (Baker's yeast)	167
Rattus norvegicus (Rat)	127
Bos taurus (Bovine)	58
Drosophila melanogaster (Fruit fly)	27
Sus scrofa (Pig)	26
Schizosaccharomyces pombe (strain 972 / ATCC 24843) (Fission yeast)	21
Caenorhabditis elegans	11
Gallus gallus (Chicken)	9
Neurospora crassa (strain ATCC 24698 / 74-OR23-1A / CBS 708.71 / DSM 1257 / FGSC 987)	9
Trypanosoma brucei brucei	8
Trypanosoma brucei brucei (strain 927/4 GUTat10.1)	7
Oryza sativa subsp. japonica (Rice)	7
Ascaris suum (Pig roundworm) (Ascaris lumbricoides)	6
Plasmodium falciparum (isolate 3D7)	6
Dictyostelium discoideum (Social amoeba)	6
Danio rerio (Zebrafish) (Brachydanio rerio)	5
Candida albicans (strain SC5314 / ATCC MYA-2876) (Yeast)	4
Solanum tuberosum (Potato)	4
Emericella nidulans (strain FGSC A4 / ATCC 38163 / CBS 112.46 / NRRL 194 / M139) (Aspergillus nidulans)	3
Pisum sativum (Garden pea) (Lathyrus oleraceus)	3
Oryctolagus cuniculus (Rabbit)	3
Ovis aries (Sheep)	3
Fusarium oxysporum f. sp. lycopersici (strain 4287 / CBS 123668 / FGSC 9935 / NRRL 34936) (Fusarium vascular wilt of tomato)	2
Chaetomium thermophilum (strain DSM 1495 / CBS 144.50 / IMI 039719) (Thermochaetoides thermophila)	2
Cryptococcus neoformans var. grubii serotype A (strain H99 / ATCC 208821 / CBS 10515 / FGSC 9487) (Filobasidiella neoformans var. grubii)	2
Cricetulus griseus (Chinese hamster) (Cricetulus barabensis griseus)	2
Zea mays (Maize)	2
Columba livia (Rock dove)	2
Euglena gracilis	2
Yarrowia lipolytica (strain CLIB 122 / E 150) (Yeast) (Candida lipolytica)	2
Ostreococcus tauri	1
Wickerhamomyces anomalus (strain ATCC 58044 / CBS 1984 / NCYC 433 / NRRL Y-366-8) (Yeast) (Hansenula anomala)	1
Aspergillus fumigatus (strain CBS 144.89 / FGSC A1163 / CEA10) (Neosartorya fumigata)	1
Phaeodactylum tricornutum (strain CCAP 1055/1)	1
Antirrhinum majus (Garden snapdragon)	1
Tribolium castaneum (Red flour beetle)	1
Ectocarpus siliculosus (Brown alga) (Conferva siliculosa)	1
Pediculus humanus subsp. corporis (Body louse)	1
Equus caballus (Horse)	1
Medicago truncatula (Barrel medic) (Medicago tribuloides)	1
Oryzias latipes (Japanese rice fish) (Japanese killifish)	1
Humulus lupulus (European hop)	1
Saccharomyces cerevisiae (Baker's yeast)	1
Sauromatum venosum (Voodoo lily) (Typhonium venosum)	1
Rhodotorula graminis (Yeast)	1
Canis lupus familiaris (Dog) (Canis familiaris)	1
Chlamydomonas reinhardtii (Chlamydomonas smithii)	1
Glycine max (Soybean) (Glycine hispida)	1
Aedes aegypti (Yellowfever mosquito) (Culex aegypti)	1
Nicotiana tabacum (Common tobacco)	1
Spinacia oleracea (Spinach)	1
Leishmania major	1
Aspergillus fumigatus (strain ATCC MYA-4609 / CBS 101355 / FGSC A1100 / Af293) (Neosartorya fumigata)	1
Aspergillus fumigatus (Neosartorya fumigata)	1
Mentha piperita (Peppermint) (Mentha aquatica x Mentha spicata)	1
Kluyveromyces lactis (strain ATCC 8585 / CBS 2359 / DSM 70799 / NBRC 1267 / NRRL Y-1140 / WM37) (Yeast) (Candida sphaerica)	1
Mesocricetus auratus (Golden hamster)	1
Leishmania tarentolae (Sauroleishmania tarentolae)	1
Candida tropicalis (Yeast)	1
Chlorocebus aethiops (Green monkey) (Cercopithecus aethiops)	1
Candida glabrata (strain ATCC 2001 / BCRC 20586 / JCM 3761 / NBRC 0622 / NRRL Y-65 / CBS 138) (Yeast) (Nakaseomyces glabratus)	1
Vigna unguiculata (Cowpea)	1
