# Supplementary material for: A protein-specific priority code in presequences determines the efficiency of mitochondrial protein import
Source: PLoS Biol. 2025 Jul 21;23(7):e3003298. doi: 10.1371/journal.pbio.3003298 (PMC12306757; doi:10.1371/journal.pbio.3003298)
Supplement: Attachment — Submitted filename: Roedl_Presequences_PLOS-response-r.pdf [file pbio.3003298.s019.pdf]

Point-by-point reply to reviewer comments

Reviewer #1:

In this study, Rödl and colleagues conduct a thorough investigation into the features of mitochondrial presequences, aiming to unravel the unique mechanisms behind protein import into mitochondria. The research introduces new techniques and offers several strong contributions. However, some aspects of the data presentation and writing suggest that the paper needs refinement.

The authors propose a priority code model in which a subset of mitochondrial precursors utilizes more potent presequences to recruit cytosolic targeting factors. The paper begins with a broad perspective and an extensive dataset, which is commendable. However, as the study progresses, the sample size of presequences used to develop and support the conclusions diminishes. For instance, the cytosolic factor TOM34, which is suggested to play a key role, is shown to interact with only one presequence (OXA1), but the conclusion is broad suggesting all strong presequences are using TOM34. This limitation undermines the findings of the work.

We thank the referee for these very positive comments and the constructive criticism. We addressed the very valid statement about a potential problem of over-generalization of the experimental data both by amendments of the text and by additional experimental data, as described in the following.

Some more specific comments can be found below.

[1] The study presents an elegant machine learning approach to categorize mitochondrial presequences, successfully identifying seven distinct groups (A-G). However, in Figure 2, the authors analyze the features of nine proteins shown in Figure 1B, but they do not select representatives from each of the proposed categories (with categories D, F, and G notably absent). This omission diminishes the perceived significance of the machine learning approach, as it appears the authors are simply using the precursors they had before conducting the categorization. Would make sense to include precursor proteins across their identified categories.

In our manuscript, we omitted the analysis of members of groups D, F and G because we wanted to include several representatives for the analyzed groups in order to determine the key properties consistently. While we are aware that selecting two to four members per group still leads to small datasets, it conclusively demonstrated that all members of group A (Oxa1, Mdl2, Pim1, also Su9) were consistently imported very efficiently. Moreover, the groups D, F and G contain many proteins that are poorly characterized, have unconventional targeting signals and were found to be dually localized to mitochondria as well as to other compartments. Thus, these signals might be more ambiguous, for example to send proteins to more than one compartment. This is certainly an interesting aspect, but we felt that it would be beyond the scope of this study to follow this up. We now added a better explanation to the text to clarify why we focus specifically on the groups which we had further analyzed.
However, we also addressed the important critique of being too narrow in several ways (see also above): (1) we tested whether the seven different presequence groups defined on basis of their structure were also physiologically relevant on basis of published proteomic datasets and found that they indeed were (novel Fig. S1C); to this end we tested whether the representatives of the groups showed a consistent patterns upon different import conditions and found that they did in several cases; (2) we analyzed the relevance of Cns1 on the mitochondrial (and cellular) proteome unambiguously, showing that this co-chaperone is of particular importance for mitochondrial proteins; (3) on basis of this dataset, we analyzed the Cns1-dependence of the seven different presequence groups which showed that group F is particularly affected by Cns1 depletion – proteins of the mitochondrial ribosome are enriched in group E as well as the Cns1-dependent proteins; and (4) we experimentally tested the binding of purified TOMM34 to peptides of sequences that include now all seven groups of presequences. We are very grateful for the

encouragement to explore the relevance of the seven types of presequence more comprehensively and will certainly continue the characterization in the future.

[2] The precursors suggested to have stronger import capabilities (Oxa1, Su9, Mdl2, and Pim1) also possess longer presequences. On page 4, it is stated that "presequences of this group carry structural determinants that endow them with an increased import competence." Could this simply be attributed to the length of the presequences? The authors should explore this possibility by categorizing the precursors identified in their machine learning approach according to presequence length and investigating import efficiency as a correlation of presequence length.

This is an interesting point. We indeed found that the ‘more potent’ presequences were typically longer. In order to test whether length per se leads to a more efficient import, we generated two test proteins in which we had different repeats of the same minimal targeting sequences derived from the N-termini of the mitochondrial proteins Atp1 and Mae1 (both having the highest TargetP scores among all mitochondrial proteins in yeast). We generated fusion proteins consisting of 0.5, 1, 2 or 4 copies of these presequences in tandem and tested their import efficiency on basis of their processing in the in vitro import assay. As shown in the Figure 1 in this letter, we found that the import efficiency did not correlate with length and the overall amount of the processed proteins remained rather constant. This suggests that it is not just a longer presequence that makes the difference but rather the presence of elements in these sequences, potentially for binding of targeting factors such as Cns1 or TOMM34. This is also supported by the peptide spot assay now shown as novel Fig. 6F which identified a TOMM34-binding sequence in the presequence of Oxa1.
We will certainly follow up the analysis of the relevance of the length of presequences in the future. Since we felt that our understanding of this aspect is still too preliminary to include it in our study, we only show this experiment here in the letter for inspection by the referee.

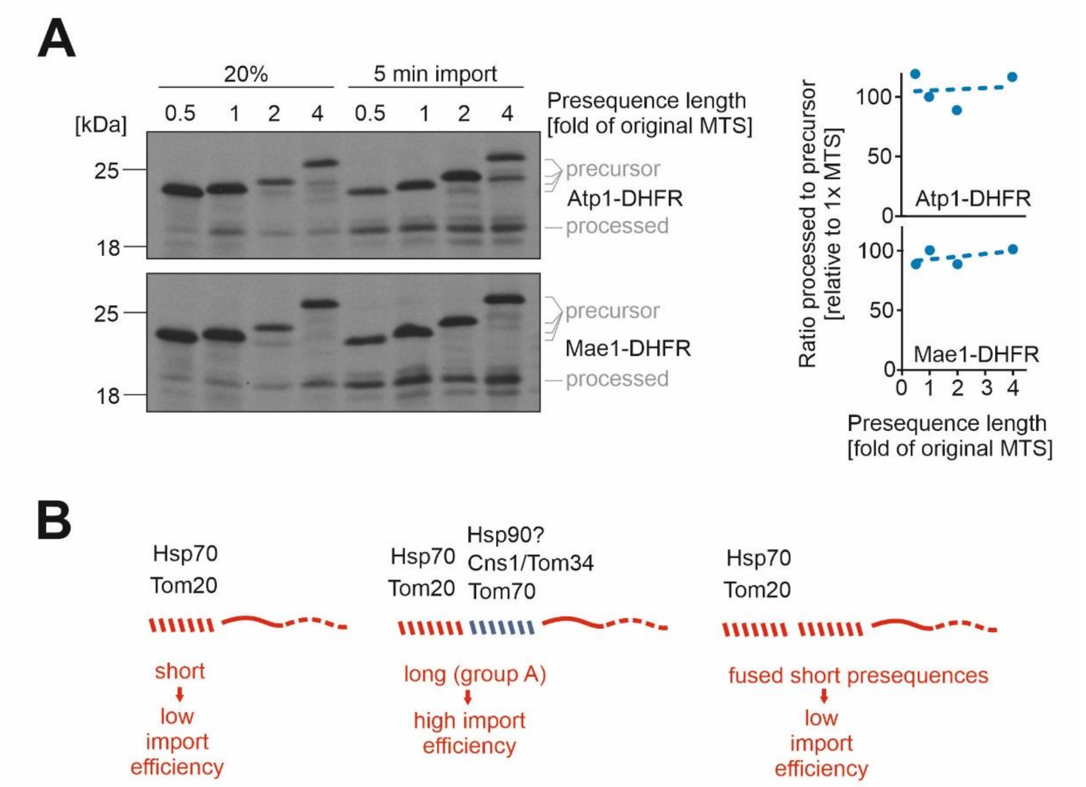

Figure 1: The import efficiency of precursors does not solely rely on the presequence length. (A) DHFR fused to either the physiological presequence or variants consisting of either half, double or four presequences in tandem were synthesized as radiolabeled precursors and incubated with isolated mitochondria for 5 min at 30°C. (B) The amounts of processed mature proteins relative to the precursor forms were shown in relation to the value for the protein containing the presequence once.

[3] The conclusions drawn from Figure 2 and Supplementary Figure 2 need to be toned down. The authors state on page 5 "The import efficiency of all proteins into these compromised mitochondria was consistently lower (Fig. 2E, F, S2B, C)." However, while the atp5 mutant shows a broad defect, the cox18 mutant is less pronounced. There is minimal quantification for these experiments and the authors should therefore moderate the language used in these conclusions and provide quantifications for some precursors across the different categories to support their claims.

We changed the wording of the respective section as suggested and added a quantification to Figs. S2B and S2C (now S3B and S3C).

[4] Pre-incubation experiment (Fig 5C and D) where it is concluded: longer preincubations do not affect the import of Atp5-DHFR, but strongly that of Oxa1-DHFR (Fig. 5C, D), suggesting that the cytosolic factor might be released upon prolonged incubation in the buffer. Can the authors show that this is not due to the OXA1 precursor aggregating and coming out of solution and there is therefore less protein available for import.

We followed the suggestion of the referee and tested the aggregation behavior of Oxa1-DHFR. As shown in the novel Figs. 5E, F, 7A, B, this precursor indeed becomes insoluble upon dilution in import buffer over time which is repressed by addition of 30 mg/ml BSA or 3 mg/ml TOMM34. Interestingly, TOMM34 but not BSA improved the import efficiency of Oxa1-DHFR indicating that the relevance of TOMM34 is not simply explained by its potential to prevent precursor aggregation.

[5] The relevance of Figure 5 and just looking at ATP5 and OXA1 really diminishes the significance of the paper. If the conclusions of this section are to be taken seriously it need to be assessed across a larger spectrum of substrates.

The results shown in Figures 5 and 6 demonstrate that different presequences differ in their dependence on TOMM34, using here the two extreme cases Atp5 and Oxa1 as model proteins. We agree with the referee that from these data, it is not possible to estimate how many presequences (and precursor proteins) depend on such cytosolic co-chaperones with TPR structure. In order to test this, we added a novel data set into the novel Fig. 8 of the study in which we measured the proteomes of cells in which Cns1, the TOMM34 homolog of yeast, was depleted. These experiments indicated that Cns1 is particularly important for subunits of the mitochondrial ribosome which are characterized by presequences of group F (novel Fig. 8G). Thereby, the Cns1-dependence differed between different presequence classes. The binding of Cns1 to the precursors in the cytosol is presumably not restricted to the presequences. It will be exciting to study the specific molecular role in Cns1 in the future which arguably might modulate the interplay of precursors, Hsp70, Hsp90 and Tom70. We thank the referee for the suggestion, to add a more general dataset to our study and hope that these additional experiments address the criticism adequately.

[6] Tom34 enrichment with OXA1. It would be good to assess the relevance of this finding to the overall picture of this study. Is this an OXA1 specific phenotype, or something specific for precursors with longer presequences. This experiment should be undertaken with some stronger and weaker presequences to support the authors claims.

We addressed this point by showing the results from peptide spot assays which confirmed the strong interaction of the Oxa1 presequence with TOMM34. We also tested the binding to other presequences. While many presequences bound TOMM34 to some degree, Oxa1 contained a region with particularly high affinity for TOMM34. These data are shown as novel Figs. 6F and S5G.

[7] The results and discussion sections include speculative conclusions about Cns1 functioning similarly to Tom34 in yeast, despite the fact that Tom34 could not rescue the growth phenotype

associated with Cns1 deficiency in yeast. This speculation regarding Cns1 should be tempered in the discussion. The authors should consider synthesizing the OXA1 presequence, along with appropriate controls, in yeast translation extracts to directly assess whether Cns1 interacts with these presequences.

We followed the suggestion of the referee and rewrote parts of the discussion. Moreover, we performed a proteomics experiment in which we compared the mitochondrial proteome of Cns1-depleted cells with or without expression of the human TOMM34 protein. This clearly demonstrated that TOMM34 does not suppress the defects of Cns1-deficient cells (novel Figure S6C-E). Even though TOMM34 and Cns1 are structurally similar and share domains with tetratricopeptide repeats (novel Fig. 8A), TOMM34 cannot replace Cns1 here. Since these co-chaperones presumably coordinate the activity of several factors, including the complex system of the Hsp90 folding machinery, it is not surprising that they cannot be easily exchanged.
We agree with the referee that it would be interesting in the future to make use of a yeast-based translation system, in which the specific relevance of cytosolic proteins (Ssa1-4, Ydj1, Cns1, Hsp82) could be studied individually. However, we felt that this is beyond this study.

Reviewer #2:

Many mitochondrial proteins contain an N-terminal cleavable presequence that carries a targeting signal for the mitochondrial matrix. Although these presequences generally form a positively charged amphiphilic helix, which functions as the mitochondrial targeting signal, they vary in length, amino-acid sequences, and targeting/import efficiency. In this study, Rödl et al. conducted in-silico analyses of mitochondrial presequences and successfully classified them into several categories (Groups A to G), each exhibiting different import efficiencies. Subsequently, they performed both in vitro and newly developed in vivo import assays to investigate the reasons behind these variations in import efficiency. They found that presequences of Group A proteins, such as Oxa1, exhibit higher import efficiency, prompting further investigation into the underlying mechanisms. Specifically, they sought to uncover the mechanism responsible for the higher import efficiency observed in Group A precursors. This study offers insights into the longstanding question of presequence properties, potentially uncovering previously elusive mechanisms that regulate import efficiency. However, the authors' interpretation of the reasons for the different import efficiencies among the various groups of presequences lacks sufficient experimental evidence. Consequently, the manuscript's current content is not compelling enough to convince me and appears too premature for publication.

We are pleased to read that the referee feels that the topic is interesting and relevant. We addressed the points raised as described in the following.

1. The authors found that the differences in import efficiency among the different presequence groups were not affected by reduced energization, i.e. by the lowered membrane potential across the inner membrane or decreased ATP levels in the mutant cells such as delta-cox18 or delta-atp6 cells (Fig. 2E and F). Based on these observations, the authors concluded that "the import efficiency differences are not due to different intramitochondrial requirements but presumably by a step upstream, i.e. by the targeting to and interaction with mitochondrial surface receptors" (page 5). However, this conclusion has shortcomings. It is already known, in contrast to the interpretation here, that different presequeces can be affected by different levels of the membrane potentials when modulated by different concentrations of CCCP (Martin et al., JBC 266, 1991). Indeed, even the Oxa1-DHFR import here looks less sensitive to the decrease in the membrane potential than Atp5-DHFR (Fig. 2E).
Therefore, the authors should perform in vitro import assays with CCCP titration to validate their findings.

This point is related to comment [3] of referee 1. As already described above, we changed the text and added a quantification. This shows, as already pointed out by the referee, that some

presequences are more affected than other when the membrane potential drops. Indeed, it was shown in several previous studies (including the one of Martin et al, cited by the referee) that titration of CCCP affects different precursor proteins to different degrees. It is likely that we would see the same for our model substrates which only differ in their presequences. Nevertheless, the point we want to make here is that despite these differences, the ‘strong‘ presequences remained ‘strong’ and the overall order did not considerably change. This is also obvious now from the quantification (novel Fig. S3B, C). This is why we concluded that the properties of the presequences affect the targeting to the TOM complex, in addition to the intramitochondrial import through the TOM and TIM23 complex. We now explain this better in the revised version of the study.

2. A potential problem the authors should address is the complexity of the protein import process, which involves multiple steps and various factors and mechanisms. The authors state that "these factors might support the import in two ways: first, by increasing the targeting efficiency to the mitochondrial surface, and second, by the unfolding of precursor proteins to facilitate their translocation through the mitochondrial protein translocases" (page 8). However, this statement is speculative and lacks experimental support, necessitating further experimental assessment. If the former is correct, the targeting step should be the rate-limiting one in the overall import process, which could be experimentally confirmed through import assays using reduced amounts of isolated mitochondria (Lithgow and Schatz (1995) J.Biol.Chem.270,14267-14269). Alternatively, if the latter is the case, the folding state of precursor proteins in complex with chaperones could be tested through protease digestion analysis.

As the referee correctly states, such in vitro experiments had been performed in depth, for example comparing the import efficiency with proteins of different folding resp. unfolding properties. In the in vitro assay, both the time for targeting and for the unfolding on the surface influence the import efficiency, while the relevance of such parameters in vivo is less well understood. This is why we set out to develop an in vivo assay that monitors import efficiency under phycological conditions in whole cells.

3. They showed that the significant difference in the import efficiency between Oxa1-DHFR and other precursors was eliminated by urea denaturation of the precursor proteins prior to the import. However, since urea could affect the import machinery, the authors should try other conditions such as performing the in vitro import at a higher temperature (e.g. 37C), where the folded DHFR domain becomes more unstable, and a destabilized DHFR mutant as a passenger protein.

In this experiment, proteins were precipitated by sodium acetate and denatured by urea. These denatured proteins were diluted 200-fold in the import reaction. Thus, the mitochondria were only exposed to 40 mM urea. This is an established assay in the field at which mitochondria stay fully import competent (e.g. Eilers, Hwang, Schatz. 1988. EMBO J. 7, 1139f; Pfanner et al. 1987. Eur J Biochem. 169, 289f).
To follow the suggestion of the referee, we carried out import reactions in vivo at 37°C (and 25°C) which confirmed the high import properties of the Oxa1 presequence regardless of the temperature. These data are shown here as Fig. 2 for inspection of the referee as we felt that they are not of immediate relevance to our study here.

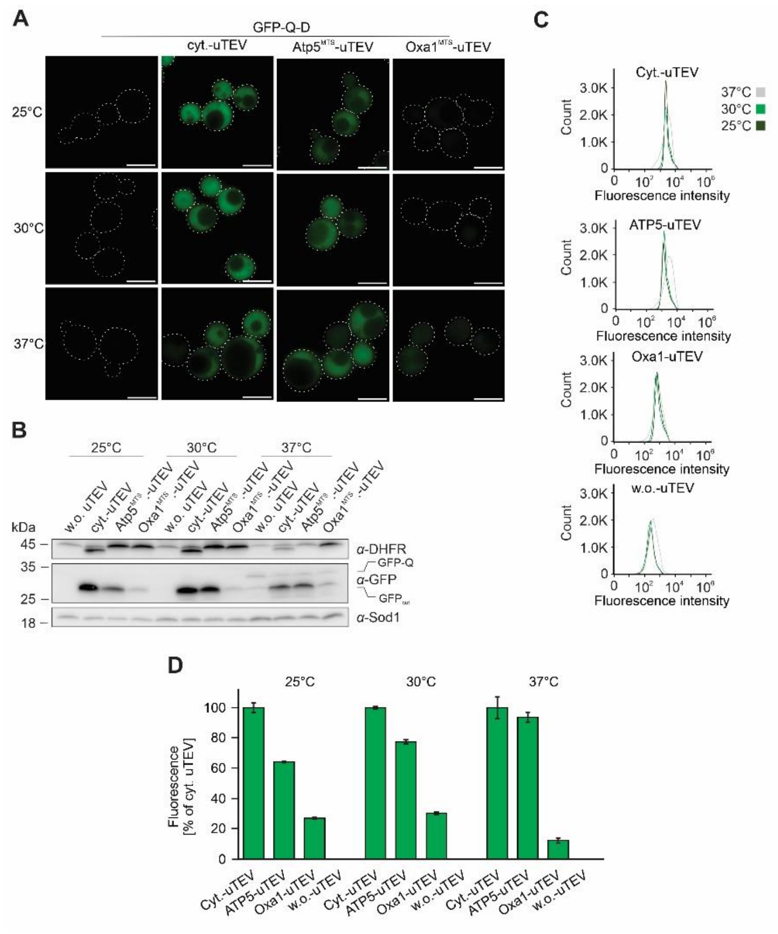

Figure 2: The Oxa1 presequence shows much higher import efficiency than that of Atp5 regardless of the temperature. We used the IQ-compete assay to quantify import efficiency of Atp5- and Oxa1-mediated import into mitochondria at 25, 30 and 37°C. The import efficiencies were analyzed by fluorescence microscopy (A), Western blotting (B), flow cytometry (C) and plate reader-based spectroscopy (D). At all temperatures, the Oxa1-uTEV protein was efficiently removed from the cytosol whereas Atp5-uTEV remained partially in the cytosol, yielding in dequenching of the GFP-Q-D construct.

4. The authors also observed that prolonged preincubation of in vitro synthesized precursor proteins after dilution into the import buffer resulted in reduced import efficiency. They speculated that a cytosolic import-stimulating factor may be released during prolonged incubation in the buffer (Fig. 5C and D). However, this experiment requires a control involving prolonged incubation without dilution or dilution into a buffer containing BSA or glycerol to mimic the cytosol crowding conditions.

We now carried out the experiment in the presence of BSA as suggested and found that BSA addition does not considerably support the import of Oxa1-DHFR even though it prevents aggregation of the protein. In contrast, upon addition of purified TOMM34 Oxa1 remained import-competent, confirming the critical role of TOMM34 as stimulating factor. This is now shown in the novel Fig. 5E, F, 7A, B. See also our response to question 4 of referee 1.

5. The authors synthesized Oxa1-DHFR and other precursors in reticulocyte lysate, followed by immunoprecipitation using anti-DHFR antibodies, and analyzed the co-precipitated proteins by mass spectrometry. They identified several cytosolic chaperones, such as Hsp70 and its co-chaperones, but found that TOMM34 co-immunoprecipitated specifically with Oxa1-DHFR and not with Atp5-DHFR (Fig. 6C). Pretreatment with anti-TOMM34 antibodies reduced the import efficiency of Oxa1-DHFR but had no effect on Atp5-DHFR (Fig. 6F). Based on these results, the authors speculated that Oxa1-DHFR binds to the cytosolic chaperone TOMM34, which could be a factor contributing its enhanced import efficiency (Fig. 6J). However, the roles of cytosolic chaperone or targeting factors are complex, as these proteins may interact with substrates transiently and in a redundant, multivalent manner. Several critical questions remain unanswered: what is the apparent MW of Oxa1-DHFR interacting with chaperones in reticulocyte lysate? How might nucleotide conditions (e.g. ATP depletion) and temperature affect the spectrum of proteins interacting with the model precursor proteins used in this study? What is the time course of import competency and possible aggregate formation for Oxa1-DHFR in reticulocyte lysate? The most critical point is that pretreatment of Oxa1-DHFR with anti-TOMM34 antibodies alone is insufficient

to demonstrate that TOMM34 is the primary factor responsible for the higher import efficiency of Oxa1-DHFR. The authors should first deplete TOMM34 from the reticulocyte lysate using anti-TOMM34 antibodies, and then synthesize Oxa1-DHFR to test its import efficiency under these conditions.

We fully agree with the referee that cytosolic proteins may interact with substrates transiently and in a redundant, multivalent manner. TOMM34 is only one of several players here. In vitro studies have shown that TOMM34 (and Cns1 as well) facilitates the transfer of proteins from Hsp70 to Hsp90. Thereby, TOMM34/Cns1 do not bind ATP, but of course both Hsp70 and Hsp90 are ATP-regulated. It will be interesting to characterize the interplay of these factors with precursors and with Tom70 in depth, but we feel that this is beyond this study.

6. They found that the import of Oxa1-DHFR was less efficient in the absence of Tom70, which functions as a docking point for the Hsp70 and Hsp90 family proteins for import. However, as Tom70 itself also has chaperone functions (Yamamoto et al., JBC 284, 31635 (2009)), Tom70 may directly interact with the Oxa1 presequence to prevent its aggregation. This possibility should be tested experimentally.

Again, the interplay of Hsp70, Hsp90, cytosolic cochaperones and Tom70 needs to be studied in depth. We are not convinced that a binding assay of Oxa1-DHFR to isolated Tom70-GST adds much to our study. Similar experiments had been carried out with other precursors (e.g. Atp1) and showed the direct binding of peptides of the presequence to Tom70 (Backes et al. 2018 JCB 217, 1369f).

Other points
The newly proposed GFP-based in vivo import assay is interesting, but it lacks kinetic information, making it unclear which step of the import process is rate-limiting. These in vivo import assays require further characterization to be efficiently compared with in vitro import results.

The in vivo assay does not reveal the import kinetics but is an ‘endpoint assay’ which monitors the efficiency of the import reaction. We agree that this is not necessarily the same as the import rate even though both parameters often correlate. We therefore compare in our study the results of the in vitro import reaction and the in vivo import reaction and were pleased to see that they strongly correlated (Fig. 4E). We therefore concluded that the model proteins that slowly imported in vitro were imported with lower efficiency in vivo.

Line12 in page 6 - group 1 should read group A

We corrected this.

Reviewer #3:

Mitochondrial protein biogenesis depends on the import of precursor proteins from the cytosol. Most of the precursors carry N-terminal extensions (presequences), which are cleavable targeting signals that mediate targeting to the organelle and subsequent translocation across the mitochondrial membranes. While presequences have been identified using N-proteomic approaches, a global analysis of their characteristics has not been performed so far. In the present manuscript, Rödl et al set out to fill this knowledge gap using machine learning and different import assays.

The analysis of presequence characteristics as the major mitochondrial targeting signals is of interest to the field of protein localization, and the current manuscript provides an interesting technical approach to study mitochondria protein targeting in vivo.

We thank the referee for these supportive comments.

However, the results obtained are preliminary and do not provide new biological insight. This is mainly due to the fact that the manuscript is not consistent as it seems that different ideas and data have been forced together. I have three main criticisms:

The machine learning approach yields 7 classes. While one would expect the authors to proceed with the analysis of 1-2 representatives of these new classes, they have chosen to continue with the classical presequences used before (by this they cover only four of the "new" classes), ignoring their own findings. It is therefore not clear why the machine learning approach was carried out at all.

This comment is almost identical to point 1 of referee 1. The same answer applies here: In our manuscript, we omitted the analysis of members of groups D, F and G because we wanted to include several representatives for the analyzed groups in order to determine the key properties consistently. While we are aware that selecting two to four members per group still leads to small datasets, it conclusively demonstrated that all members of group A (Oxa1, Mdl2, Pim1, also Su9) were consistently imported very efficiently. Moreover, the groups D, F and G contain many proteins that are poorly characterized, have unconventional targeting signals and were found to be dually localized to mitochondria as well as to other compartments. Thus, these signals might be more ambiguous, for example to send proteins to more than one compartment. This is certainly an interesting aspect, but we felt that it would be beyond this study to follow this up. We now added a better explanation to the text to better explain why we focus specifically on the groups which we had further analyzed.
However, following up on this criticism we now deepened our analysis of the different groups of precursors. To this end, we analyzed several different published datasets in which import efficiencies were reduced for different reasons (absence of chaperones, mutations in import components, competitions with cloggers) or in which the co- vs post-translational import rates were quantified (novel Fig. S1C). This showed that the seven different groups show clearly group-specific characteristics, supporting our conclusion that presequences influence the biogenesis of mitochondrial precursors in complex ways.

The main finding of the manuscript is the requirement of TOMM34 for a specific precursor protein. TOMM34 was identified using a proteomics approach. Unfortunately, it is not clear why the authors focused on TOMM34, as many other proteins show a stronger enrichment (the proteomics data are only available on PRIDE, which is not user-friendly, and usually additional supplemental excel files are provided for easy assessment and evaluation of the data). While the choice of TOMM34 is not sufficiently clear, the real problem lies in the whole experimental set-up: The authors use isolated yeast mitochondria, but combine them with precursor proteins synthesized in rabbit reticulocyte lysate. It is true that this approach has been used for decades in the mitochondrial protein biogenesis field. While this may be acceptable for the study of intramitochondrial translocation processes, it is highly problematic when investigating the early cytosolic steps of mitochondrial protein targeting. Yeast extract for in vitro translation is well established and has been successfully used by other groups to study cytosolic events in mitochondrial targeting (in particular by the Rapaport lab). Here, using a rabbit translation system, the authors identify TOMM34 and assign it a role in mitochondrial targeting, despite the fact that yeast does not have a TOMM34 homologue. Therefore, there are no data on the in vivo relevance of this protein.

Cns1 is a cytosolic cochaperone which similar TPR structure as TOMM34. Like TOMM34, it facilitates the Hsp70-to-Hsp90 transfer of substrate proteins (Schopf et al. 2019 Mol Cell 74, 74f). Inspired by the question about in vivo data in yeast, we analyzed the proteome of Cns1-depleted and wild type cells. The results are shown in the newly added Fig. 8 where we found that many mitochondrial proteins were depleted upon loss of Cns1 (novel Figures 8E-G, S6A-D). We are grateful for this suggestion which clearly improved our study.

Moreover, we fully agree with the referee that the reticulocyte lysate approach has limitations. This is why we developed the new in vivo assay which is introduced in this study. We are convinced that this assay is very valuable.

Finally, the authors identify the presequence of Oxa1 as the most efficient. This is curious as the group has previously published a novel protein import pathway, in which precursors are imported into mitochondria via the ER (Hansen et al., ER SURF pathway). If the import of Oxa1 is the fastest, how and why would the precursor then be re-routed via the ER? Unfortunately, the authors do not mention the ER-SURF at all in the manuscript, which is surprising given that they identified this new import pathway.

We now added a section to the discussion in which we mention the ER-SURF pathway. Oxa1 is a rather abundant and an important inner membrane protein. It has five hydrophobic transmembrane domains. Eukaryotic cells improved its import by several tricks, such as ER-SURF targeting (Hansen et al. 2018 Science 361, 1118f), proline residues in the transmembrane domains (Meier et al. 2005 JCB 170, 881f), Puf3-binding segments in the 5'-UTR of its mRNA (Sylvestre et al. 2003 MBoC 14, 3848) and a very powerful presequence (this study). We can only speculate why Nature developed these strategies to improve the import of Oxa1 but it seems likely that these adaptations allowed it to express this hydrophobic protein from the nuclear rather than from the mitochondrial genome. It should be mentioned that others of the efficient presequences we found are also used for hydrophobic proteins (e.g. Mdl2, an ABC transporter of the inner membrane with even six transmembrane domains that was shown to be a complicated substrate of the import machinery; Bohnert et al. 2010. Current Biol. 20, 1227f).
